# Microbiome of High-Rank Coal Reservoirs in the High-Production Areas of the Southern Qinshui Basin

**DOI:** 10.3390/microorganisms11020497

**Published:** 2023-02-16

**Authors:** Wei Shi, Shuheng Tang, Songhang Zhang

**Affiliations:** 1 MOE Key Lab of Marine Reservoir Evolution and Hydrocarbon Enrichment Mechanism, Beijing 100083, China; 2 MOLR Key Lab of Shale Gas Resources Survey and Strategic Evaluation, Beijing 100083, China; 3 School of Energy Resources, China University of Geosciences (Beijing), Beijing 100083, China

**Keywords:** coalbed methane, hydraulic zones, C-N-S microorganisms, southern Qinshui Basin

## Abstract

To study the distribution features of microorganisms in distinct hydrological areas of the southern Qinshui Basin, C-N-S microorganisms were studied using 16S RNA sequencing, metagenome sequencing and geochemical technologies, showing the high sensitivity of microorganisms to the hydrodynamic dynamics of coal. The hydrodynamic intensity of the #3 coal gradually decreased from the runoff areas to the stagnant areas. The stagnant zones have higher reservoir pressure, methane content, δ^13^C_DIC_ and TDS and lower SO_4_^2−^, Fe^3+^ and NO_3_^−^ concentrations than the runoff areas. C-N-S-cycling microorganisms, including those engaged in methanogenesis, nitrate respiration, fermentation, nitrate reduction, dark oxidation of sulfur compounds, sulfate respiration, iron respiration, chlorate reduction, aromatic compound degradation, denitrification, ammonification and nitrogen fixation, were more abundant in the stagnant areas. The relative abundance of C-N-S functional genes, including genes related to C metabolism (e.g., *mcr*, *mer, mtr, fwd* and *mtd*), N metabolism (e.g., *nifDKH*, *nirK*, *narGHI*, *nosZ*, *amoB*, *norC* and *napAB*) and sulfur metabolism (e.g., *dsrAB* and *PAPSS*), increased in the stagnant zones, indicating that there was active microbiological C-N-S cycling in the stagnant areas. The degradation and fermentation of terrestrial plant organic carbon and coal seam organic matter could provide substrates for methanogens, while nitrogen fixation and nitrification can provide nitrogen for methanogens, which are all favorable factors for stronger methanogenesis in stagnant areas. The coal in the study area is currently in the secondary biogenic gas generation stage because of the rising of the strata, which recharges atmospheric precipitation. The random forest model shows that the abundance of C-N-S microorganisms and genes could be used to distinguish different hydrological zones in coal reservoirs. Since stagnant zones are usually high-gas-bearing zones and high-production areas of CBM exploration, these microbiological indicators can be used as effective parameters to identify high-production-potential zones. In addition, nitrate respiration and sulfate respiration microorganisms consumed NO_3_^−^ and SO_4_^2−^, causing a decrease in the content of these two ions in the stagnant areas.

## 1. Introduction

Nowadays, the world is facing an energy shortage, and the exploration and exploitation of fossil fuels are important research topics [1]. Coalbed methane (CBM) is a crucial clean energy source and is rich in reserves in coal-bearing basins [2]. In addition, a coal seam has strong adsorption for CO_2_ and is an ideal medium for CO_2_ burial and conversion. An important topic in the process of CBM exploration and development is the search for high-gas-bearing areas, where the screening parameters include CBM gas content, total dissolved solids (TDS) and other hydrochemical parameters [3,4,5,6]. In this study, we innovatively applied C-N-S microbial sequencing in coal seams for the delineation of favorable CBM zones, and we achieved good results. The distribution of microorganisms in the hydraulic zones of different coal seams is more sensitive than the hydrochemical parameters and corresponds well with the measured gas content and gas production of coal seams. The research results provide an effective basis for guiding the exploration of favorable CBM blocks.

The main genetic types of CBM include thermogenic gas, biogenic gas and mixed genetic gas. Biogenic coalbed methane mainly occurs in middle- and low-rank coal, while thermogenic methane and mixed genetic methane mainly exist in high-rank coal. Microorganisms play a significant role in CBM generation and CO_2_ storage. Studying the distribution features of microorganisms and microbial species composition has the following important significance for increasing CBM production:

(1) Microorganisms in coal-bearing basins have been widely studied around the world, including in the Illinois Basin [7], the coal seams in Japan [8], the Ordos Basin [9], and the Gippsland Basin [10]. These studies used 16S rRNA sequencing technologies to analyze the microbial characteristics in coal seams. However, studies focusing on the distribution characteristics of microorganisms in different hydrologic zones of coal basins are deficient. Previous research found typical C-N-S microorganism functional genes in the produced water of CBM production wells, indicating that an active C-N-S biogeochemical cycle and possible biogenic gas may exist in the southern Qinshui Basin [11]. As hydrogeological conditions are the important reasons for the enrichment or escape of CBM in the basin, groundwater dynamics and chemical fields control the accumulation characteristics of methane. Studying the distribution characteristics of microorganisms from the perspective of species in different hydraulic zones of the Qinshui Basin is crucial for expanding knowledge of geochemical responses and biogeochemical cycles in the region.

(2) The mechanisms of biological cycling in coal are useful to guide CBM exploration [12,13,14,15]. C-N-S microorganisms, especially anaerobic respiration microorganisms, such as methanogens, nitrogen fixation bacteria, AOA, AOB, NOB and denitrifying bacteria, control the accumulation of biogenic gas in coal reservoirs [9,16,17]. They can be used as an important index to distinguish the redox zone of coal reservoirs in the basin due to their sensitivity to oxygen. Generally, the reduction environment in the hydrological stagnant areas is not only a favorable condition for CBM preservation but also a high-gas-bearing zone with high production potential. Therefore, determining the microbial distribution characteristics in a coalbed methane block is significant for finding CBM enrichment zones and high-production areas in CBM basins.

(3) Generally, when studying the functional types of microorganisms, the bacteria and archaea measured by 16S rRNA in one area are compared with those known for functions in other environments. However, there have been few studies on the classification based on the function of strains, especially the classification of C-N-S functional microorganisms. Therefore, in this study, according to 16S rRNA sequencing, the measured OTUs were systematically divided into 90 functional types, including methanotrophy, methanogenesis, aerobic ammonia oxidation, nitrogen fixation, denitrification, nitrification, dark sulfide oxidation, sulfate respiration, anammox, chemoheterotrophy and fermentation.

Using 16S rRNA sequencing, metagenome sequencing and geochemical technologies, this study assesses the distribution changes of the microorganisms in different hydrogeochemical areas in the Shizhuangnan block which is located in the southern Qinshui Basin. The Shizhuangnan block, which has more than 900 CBM wells, is an important high-yield CBM development block in Shanxi [18,19].

## 2. Geological and Hydrogeological Conditions

**Figure 1:**
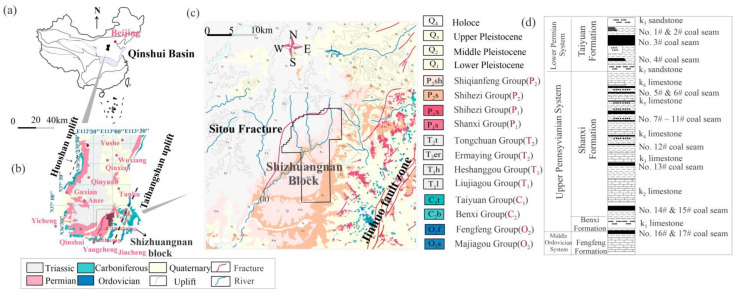
Comprehensive geological map of the study area: (**a**) location of Qinshui Basin in China; (**b**) geological map of exposed strata and location of the Shizhuangnan block in the southern Qinshui Basin; (**c**) geological map of exposed strata in the Shizhuangnan block; (**d**) stratigraphic column of the study area.

The southern Qinshui Basin is located in North China (Figure 1a) [20]. The #3 and #15 coal seams are the main CBM-producing coal. In the Shizhuangnan block, most CBM wells target the #3 coal. The #3 coal’s elevation is between 200 and 600 m (Figure S1a), and the average thickness of the #3 coal is 6 m (Figure S1d). The depth of the #3 coal is between 650 and 1000 m, and the burial depth in the northern part is more than 1000 m. The R^o^_max_ of the #3 coal is 3% (Figure S1c), and the gas content is between 10 and 30 m^3^/t (Figure S1b). The well’s average gas production is more than 500 m^3^/day. Most high-production wells are distributed in the low-lying area in the core of the western syncline, with gas production of over 1500 m^3^/day [21].

### 2.1. Main Strata

The strata that are exposed in the Shizhuangnan block include Permian, Triassic and Ordovician (Figure 1b–d) [22]. The layers that contain coal are mainly the Taiyuan Formation and Shanxi Formation (Figure 1d). The strata cropping out in the study region include the O_2_s, O_2_f, C_2_b, C_3_t, P_1_s, P_1_x, P_2_s, P_2_sh, T_1_l, T_1_h, T_2_er, T_2_t, Q_1_, Q_2_, Q_3_ and Q_4_ deposits (Figure 1c,d).

The Taiyuan Formation contains the #15 coal, and the Shanxi Formation contains the #3 coal, as shown in Figure 1d. The roof of the #3 coal is mudstone, which is continuously and stably distributed in space. The roof of the #15 coal is a regionally distributed and stable shallow marine limestone (K_2_ limestone).

### 2.2. Physical Properties of Coal Reservoirs

The coal seam is bright and dark coal in the study area, followed by specular coal. The #3 coal and #15 coal seams are the main CBM development coal seams. According to the quantitative identification results of coal macerals and minerals, the vitrinite contents in the #15 and the #3 coal seams are in the ranges of 41.15~81.15% and 51~78.3%, respectively. The contents of inertinite in the #15 and the #3 coal seams are in the ranges of 9~24.8% and 14.3~36.5%, respectively.

The ash and volatility are low in the coal seams. The variation range of ash yield in the #3 coal seam is 11.55~17.66%, and the average yield of volatile matter is 12.0%. The ash content of the #15 coal seam varies from 16.08 to 36.20%, and the yield of volatile matter is between 9.59 and 20.23%. The vitrinite reflectance (R^o^_max_) of the #3 coal seam is between 2.92 and 3.02%, and the hydrogen content (H_daf_) is between 3.99 and 4.24%. The #3 coal is classified as anthracite III coal according to the Chinese coal classification standard GB5751-86.

The reservoir pressure of the #3 coal is 1.75~6.14 MPa, and the critical desorption pressure is 0.7~3.32 MPa. The reservoir pressure of the #15 coal is between 2.22 and 4.61 MPa, and the critical desorption pressure is 0.77~3.45 MPa.

The porosity of the #3 coal is between 4.72 and 5.96%, and that of the #15 coal seam is between 4.97 and 5.95%. The injection/pressure drop test shows that the measured permeability of the #3 coal is generally between 0.01 × 10^−3^ and 1.2 × 10^−3^ μm^2^. The permeability measured in the #15 coal seam is between 0.02 × 10^−3^ and 1 × 10^−3^ μm^2^.

For the #3 coal seam, the isothermal adsorption test results show that the dry ash-free Kirschner volume is between 33.76 and 47.16 m^3^/t, and the Kirschner pressure is between 1.94 and 3.07 MPa. For the #15 coal seam, the dry ash-free Kirschner volume is 14.27~46.37 m^3^/t, and the Kirschner pressure is between 1.97 and 3.78 MPa.

### 2.3. Gas Characteristics of the Coal Seam

According to the analysis of the preservation conditions of CBM, the coal was buried to its deepest depth in the Indosinian period, and methane was generated and adsorbed in the coal seam. The thermal event in the Yanshan period made the southern Qinshui Basin an abnormal paleogeothermal field, and its geothermal gradient could reach 4~6 °C/100 m, generating thermal methane. The methane content of the #3 and #15 coal increases from the edge to the deep basin (Figure S3). The gas saturation of the #3 coal is 49.44~85.67%, and that of the #15 coal seam is 50.79~80.96%.

According to the gas composition results in the study area, there is little difference between the gas components of the #3 and #15 coal seams. The main component is methane, which is more than 90%. The second is nitrogen, which is generally less than 5%. The carbon dioxide content is generally less than 3%. Trace amounts of heavy hydrocarbons can also be detected in some samples.

### 2.4. Hydrogeological Characteristics

The stratigraphic trend in the study area is NNE and dips toward the west. The Sitou fault is a nonconducting fault that controls the hydrodynamic and hydrochemical fields [22].

The #3 coal is topped by mudstones. The #3 coal wells’ water production is often limited. However, in the northern Shizhuangnan block, the sandstone aquifers and the #3 coal seam are connected by hydraulic conductivity faults, increasing water production.

**Figure 2:**
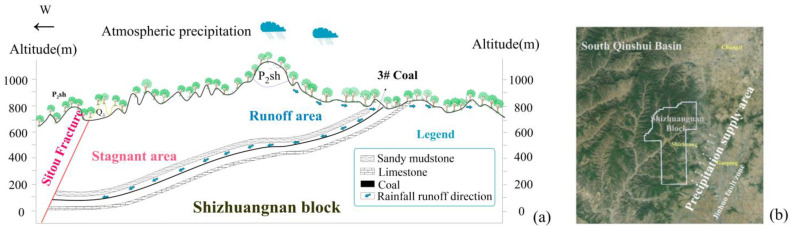
(**a**) Distribution of #3 coal seam reservoir and seepage of atmospheric precipitation in the study area; (**b**) satellite topographic map of the study area.

From east to west, the height of the #3 coal seam steadily drops (Figure 2a). In the eastern part of the research region, the monoclinic structure allows atmospheric precipitation to seep from east to the west (Figure 2a). As the coal seams extend to the west, the hydrogeological circumstances of the change from the eastern runoff to the western stagnant areas are unique because of the steady deterioration of the hydrodynamic conditions. With the decrease in dissolved oxygen in the water from the runoff areas to the stagnant areas, groundwater’s redox environment shifts from an oxidizing to a reducing one. The stagnant areas are usually high-yield areas due to their gas concentration, displaying the characteristics of water resistance and stagnation of groundwater in low-lying areas.

The variations of C-N-S microorganisms in various hydrological areas and the connection between the geochemical properties and microorganisms are the main subjects of this study. The C-H-O-N stable isotopic and ion compositions in CBM-coproduced water of the #3 coal seams were analyzed, and the microbial species were examined to assess the effects of microbes. More than 100 CBM water samples were taken from 2016 to 2021, and the average daily gas production of over 500 wells was counted. The distribution of samples in the study area is shown in Figure S2. This study mainly analyzed the 23 microbiological and geochemical test data from 2019, and the data tested in 2016 and 2018 were used for the analysis of strains, as shown in Figure S8.

## 3. Materials and Methods

### 3.1. Sample Collection

From 2016 to 2021, more than 100 water samples were collected (Figure S2a). The CBM wells have been continuously operating for more than 8 years. Water samples were taken in 50 mL centrifuge tubes and 500 mL headspace bottles straight from the CBM wellhead. Additionally, gas sampling was performed using 0.5 L bags. 

Geochemical test methods for geochemical data, including ion concentration, nitrate nitrogen and oxygen isotope, water hydrogen and oxygen isotope, dissolved inorganic carbon (DIC) isotope, and methane isotope, were based on the coal industry standard for analysis of the water quality of coal mine water (MT/T894-2000).

Using the PowerWater DNA Isolation Kit (MO BIO Laboratories, Carlsbad, CA, USA), the microbial community’s DNA was isolated in accordance with the manufacturer’s instructions. Using the Qubit dsDNA BR Assay kit (Invitrogen, USA), DNA was measured with a Qubit Fluorometer, and the quality was confirmed by running an aliquot on a 1% agarose gel.

### 3.2. Gene Sequencing Analysis

Degenerate PCR primers 341F (5′-ACTCCTACGGGAGGCAGCAG-3′) and 806R (5′-GGACTACHVGGGTWTCTAAT-3′) were used to amplify the 16S rRNA gene’s V3-V4 region. Illumina adapter, pad and linker sequences were added to the forward and reverse primers. In a 50 L reaction with 30 ng of template, fusion PCR primer and PCR master mix, PCR enrichment was carried out. PCR cycling conditions were as follows: 94 °C for 3 min, 30 cycles of 94 °C for 30 s, 56 °C for 45 s, 72 °C for 45 s and final extension for 10 min at 72 °C for 10 min. Am-pureXP beads were used to purify the PCR products, and Elution buffer was used to elute them. An Agilent 2100 bioanalyzer was to certify libraries (Agilent, USA). The verified libraries were utilized for sequencing on an Illumina HiSeq platform.

FastQC was used to assess the paired-end Illumina reads’ quality. Raw reads were filtered to remove adaptors and low-quality and ambiguous bases, and then paired-end reads were added to tags by FLASH to obtain the tags. Using the UPARSE program, the tags were clustered into OTUs with a cut-off value of 97%, and chimera sequences were compared with the Gold database using UCHIME. OTU representative sequences were taxonomically classified using the RDP Classifier and trained on the Greengenes database using QIIME v1.8.0. USEARCH was used to compare all tags with OTUs to obtain the OTU table. Alpha and beta diversity values were estimated using MOTHUR and QIIME. KEGG functions were predicted using PICRUSt. The Venn plots of OTUs were plotted with the R package “VennDiagram”. 

By comparing the names of different taxonomies to the Functional Annotation of Prokaryotic Taxa v.1.0, which was made by combining many culturable prokaryotic microorganisms with known functions, the taxonomies were classified into 90 different groups, including a typical C-N-S functional community, such as methanogenesis group, nitrification group, sulfate respiration group, denitrification group, dark sulfide oxidation group, fermentation group, aromatic compound degradation group and hydrocarbon degradation group.

To examine the differences in microbial communities across various environmental groups, three complementing nonparametric multivariate analyses (Adonis, ANOSIM and MRPP) were utilized. STAMP was used to identify the variation in C-N-S functional microorganisms. Welch’s *t*-tests were chosen for the analysis of microbial differences between the stagnant and runoff areas, with a *p*-value filter of 0.05.

We used the “randomForest” version 4.6-14 to categorize the abundances of bacterial taxa. The rfcv function used cross-validation to choose the right features. The significance of features in the categorization was demonstrated using the varImpPlot function.

### 3.3. Metagenome Sequencing Analysis

The qualified samples could be used for library preparation. After DNA extraction, 1 µg genomic DNA was randomly fragmented using a Covaris instrument. The Covaris instrument was used to break the DNA sample by ultrasonic wave, and the short DNA fragments meeting the length requirements were obtained by adjusting the instrument’s parameters. The fragments of the interrupted samples were selected using an Agencourt AMPure XP-Medium kit, so that the bands of the DNA samples were concentrated at about 300~400 bp; the quantity of purified DNA samples was detected using Qubit dsDNA HS Assay Kit 500 assays. The qualified libraries were sequenced on an Illumina HiSeq platform.

The raw data were cleaned up using SOAPnuke. Megahit was used to assemble high-quality reads. Assembled contigs with lengths of less than 300 bp were excluded. Using MetaGeneMarker, CDS values were predicted. A 95% identity cutoff was used in CD-HIT to remove redundant genes.

The protein sequences of genes were matched to the NR database using DIAMONDS with an E value 1 × 10^−5^ to obtain the taxonomy data. The MEGAN LCA method was used to determine the taxonomy annotation. The reads were aligned to the genes using Botwie2 with the default option to produce the taxonomic abundance profiles.

All predicted genes were searched in the public database using blast software v2.13.0+ (blast, E-value < 0.00001), including NR (2016-09), eggNOG (2019-04), CAZY (2019-06), COG (2014-11), Swiss-Prot (2019-07), KEGG (89.1) and CARD (3.0.3). Through comparison, the protein sequence with the highest similarity was selected and the corresponding protein function annotation was obtained.

### 3.4. Hydrodynamic Field Modeling

The #3 coal reservoir’s 3D geometric model and hydrodynamic field numerical simulation were created using Civil 3D and COMSOL, respectively. The western boundary was designated as having no water conduction because of the Sitou fracture’s impact of restricting water flow. In this study, we applied Comet 3 software to simulate the gas production of well sets in runoff and stagnant areas.

## 4. Results

### 4.1. Geochemical Results

As rock and water interact along the flow routes, the iron content and isotope features of the coproduced water from CBM are helpful in identifying hydraulic zones. The majority of the total solute in groundwater is made up of the ions such as Na^+^, HCO_3_^−^, NO_3_^−^ and SO_4_^2−^, which are often present in CBM-coproduced water. Coal water in the stagnant areas has greater TDS contents and lower Fe^3+^, NO_3_^−^ and SO_4_^2−^ contents, whereas water in runoff zones has lower TDS and higher Fe^3+^, NO_3_^−^ and SO_4_^2−^ contents.

While the Cl^−^ concentrations ranged from 30 to 1600 mg/L, the Na^+^ + K^+^ concentrations ranged from 240 to 1180 mg/L. The Ca^2+^ concentrations ranged from 0.1 to 10 mg/L, and the Mg^2+^ concentrations ranged from 0.6 to 4.3 mg/L. Trace elements had very low concentrations in coal seam water. The concentrations of Mo ranged from 5.3 to 17.7 μg/L. The As concentrations ranged from 0.12 to 1.98 μg/L. The I^−^ concentrations ranged from 0.002 to 0.1 mg/L. The Mn^2+^ concentrations ranged from 0.001 to 0.06 mg/L. The SO_3_^2−^ and S_2_O_3_^2−^ concentrations were in the ranges of 0.0003~0.42 mg/L and 0.0002~0.035 mg/L, respectively.

In the runoff areas, NO_3_^−^, SO_4_^2−^ and Fe^3+^ were more prevalent. These anaerobic electron acceptors are directly associated with anaerobic microbial respiration. The range of NO_3_^−^ concentrations was from 0.2 to 1.0 mg/L. The Fe^3+^ concentrations were between 0.3 and 5.6 mg/L. In the samples, 6.9 to 17.0 mg/L SO_4_^2−^ was present. δ^13^C_DIC_ was in the range of −12 to 35‰.

**Figure 3:**
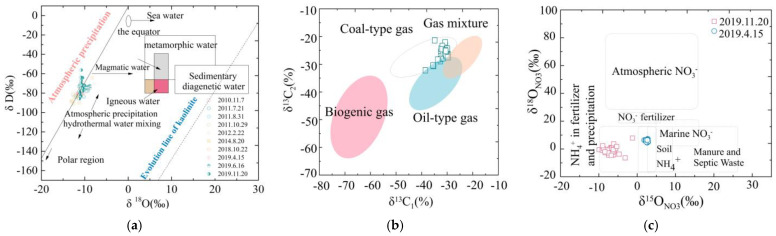
Geochemical characteristics of water samples in the study area: (**a**) hydrogen and oxygen isotopes of H_2_O (‰), (**b**) carbon isotopes of CH_4_ (‰) and (**c**) nitrogen and oxygen isotopes of nitrate (‰).

The source of groundwater may be determined using oxygen and hydrogen isotopes. The atmospheric precipitation line was calculated using the following equation: δD = 7.9δ^18^O + 8.2. Figure 3a shows that the distribution of the water samples was close to the atmospheric precipitation line, demonstrating that atmospheric precipitation was primarily the source of coal water.

The gas composition in the Shizhuangnan block was mainly hydrocarbons, including methane (64.36~95.77%) and ethane (0.001~0.018%). The only detected heavy hydrocarbon in the study area is ethane, with C_1_/C_1+2_ of 0.9998~0.9999, which is extra dry gas. The non-hydrocarbon gases include small amounts of CO_2_ (0.03~19.08%) and N_2_ (0.03~26.45%). The δ^13^C_CH4_ values were −19 to −40‰, and δD_CH4_ values were −270 to −135‰. The δ^13^C_C2H6_ values ranged from −32 to −21‰, and δD_C2H6_ values ranged from −108 to −71.64‰; gases from the study area are typical coal-type gas and have a thermogenic origin (Figure 3b). 

The R° values of the coal were 2.8~4.8%, which reach the overmature stage. The genesis type of CBM should belong to thermally cracked dry gas in the study area. 

The range of values for δ^15^N_NO_3__ and δ^18^O _NO_3__ indicates that nitrogen fixation of the NH_4_^+^ may be the source of nitrate that penetrated deep into the coal seam with atmospheric precipitation (Figure 3c).

With the stratigraphic uplift, atmospheric precipitation is currently recharging the coal seam, and conditions are in place to produce secondary biogenic gas, although gas isotopes indicate that biogenic gas is currently underrepresented. We explored the distribution characteristics of methanogenic and related C-N-S microorganisms in the runoff and stagnant hydrological areas to explore the pathways of biogas production.

### 4.2. Runoff and Stagnant Area Division

To compare the distribution disparities of C-N-S microorganisms, one of the most crucial concerns was to segment distinct hydrodynamic zones. The variables discussed below were considered.

**Figure 4:**
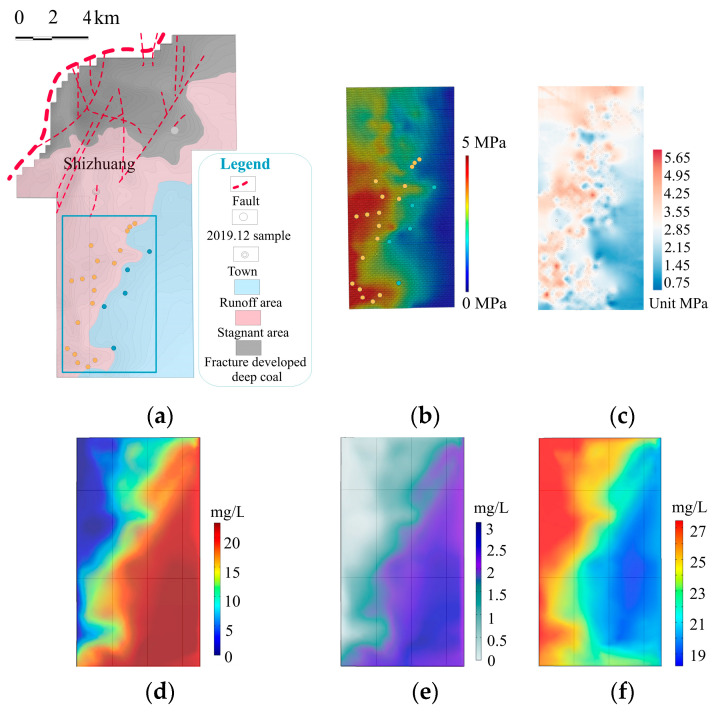
(**a**) Sample (2019.12) distribution in the study area and division between the runoff and stagnant areas; (**b**) numerical modeling of water pressure (MPa) in the sampling area within the blue box); (**c**) initial well pressure of drainage wells in the study area; (**d**) numerical modeling of SO_4_^2−^ (mg/L); (**e**) numerical modeling of NO_3_^−^ (mg/L); (**f**) numerical modeling of Ca^2+^ (mg/L).

First, a model of the sampling area’s 3D hydrodynamic field was created using Comsol because the water pressure is the direct result of the hydrodynamic field. The water pressure clearly increased rapidly in the west (Figure 4b), which was driven by the convergent water flow in the western stagnant areas because of the Sitou fracture that prevented the boundary water from flowing. This study also counted the initial bottom hole pressure of 500 drainage wells in the study area, and the contour map also verifies that the pressure increases gradually from the runoff to the stagnant areas, from 0.5 to 6.5 MPa (Figure 4c).

The black dotted line was utilized to separate the hydrodynamic zones; it represents the margin of the syncline in the western research area (Figure S3). The change in the atmospheric precipitation’s flow to a stagnant state, or the transition of runoff areas to stagnant areas, occurred over this line. It was possible to enhance and preserve coalbed methane in the stagnant environment. In fact, there was a clear variation in the methane content along this black dotted line from 7 to 20 m^3^/t (Figure S3).

**Figure 5:**
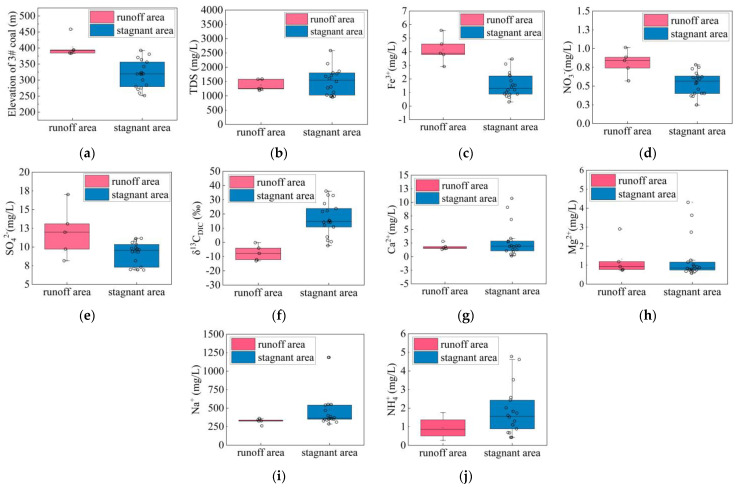
Geochemical characteristics of water ions and gas composition in runoff and stagnant areas: (**a**) #3 coal seam elevation (m); (**b**) TDS (mg/L); (**c**) Fe^3+^ (mg/L); (**d**) NO_3_^−^ (mg/L); (**e**) SO_4_^2−^ (mg/L); (**f**) δ^13^C_DIC_ (‰); (**g**) Ca^2+^ (mg/L); (**h**) Mg^2+^ (mg/L); (**i**) Na^+^ (mg/L); (**j**) NH_4_^+^ (mg/L).

The increased mineralization in the stagnant areas was corroborated by the TDS concentration (mostly Na^+^ and Cl^+^), as shown in Figure 5b, which was bolstered by the stagnant environment’s enhanced interaction between water and rock. Microorganisms’ anaerobic respiration strengthened as precipitation moved to the west stagnant areas with decreased O_2_. This led to the utilization of anaerobic electron acceptors, such as NO_3_^−^, SO_4_^2−^ and Fe^3+^, in the stagnant areas. Anaerobic electron acceptors involved in a number of significant anaerobic respiration processes, including SO_4_^2−^ reduction, denitrification and Fe^3+^ reduction, were decreased in the anoxic stagnant areas. The geochemical values changed from oxic runoff areas to anoxic stagnant areas. On both sides of this line, there was a clear difference in the concentrations of Fe^3+^, NO_3_^−^ and SO_4_^2−^ (Figure 5c–e). According to Wang et al. (2016), methanogenesis causes methane to enrich lighter carbon and deplete DIC C isotopes. The C isotopes of DIC in stationary areas would be more positive if biogenic methane production in those areas was higher. This hypothesis that microbial methanogenesis isotopically fractionated the C pool was corroborated by the findings of the dissolved inorganic carbon isotope tests (Figure 5f).

The border of the syncline was chosen to separate the runoff areas and the stagnant zones (Figure S2b), considering the structural position (Figure 2a), water pressure (Figure 4b,c), gas concentration (Figure S3) and water geochemical data (Figure 5b–f). Deep coal that was fault-developed is represented by the gray region in the north. The red area shows stagnant areas, and the blue area shows runoff areas.

The hydrodynamic circumstances were defined by the structural position, which then had an impact on the hydrochemical field distribution. According to this study, stagnant areas had higher concentrations of C-N-S microorganisms. The sequencing test supported this assumption.

### 4.3. Microbial Community Structure

Using 341F and 806R primers, 16S rRNA was amplified by PCR, and the results were utilized using Illumina sequencing, yielding 8264 operational taxonomic units (OTUs). To confirm the accuracy of the distribution features of the 16S rRNA sequencing data, metagenome sequencing was performed concurrently.

We found that the β diversity differed in runoff and stagnant samples. The Bray-Curtis, Euclidean and Jaccard distances revealed that samples from the runoff areas and stagnant areas formed two distinct clusters (Figure S4a–c). As expected, the water samples in stagnant areas differed from those in runoff areas due to the different hydrodynamic and chemical conditions.

The α diversity (Chao, Simpson and Shannon indices) showed a significant difference between runoff and stagnant area samples (Figure S6). The stagnant areas had higher diversity than the runoff areas (Figure S6a–c), indicating that stagnant areas recruited more bacterial species than runoff areas, which was also verified by the Venn diagram of microbial species in runoff areas and stagnant areas (Figure S5a) and LEfSe analysis (Figure S5b).

With increasing sequencing depth, the rarefaction curves of the alpha index of the water samples reached the saturation stage, showing that the microbiota in our test captured the majority of bacterial members from each sample (Figure S7).

For samples collected in the study area from 2016 to 2020, the species distribution is shown in Figure S8. The most prevalent bacteria at the class level were *Deltaproteobacteria*, *Alphaproteobacteria*, *Gammaproteobacteria* and *Betaproteobacteria,* which accounted for more than 85% of all microorganisms. In addition to the four most prevalent groups, *Flavobacteria*, *Clostridia*, *Bacilli*, *Bacteroidia*, *Actinobacteria* and *Methanobacteria* were also present but in considerably lower relative abundances (Figure S8).

In line with this prediction, runoff and stagnant regions have distinct functional gene structures in the microbial community. The structure of the microorganisms was different between the runoff zones and stagnant zones, according to the analysis of MRPP (Δ = 0.7, *p* = 0.004), Adonis (F = 4.65, *p* = 0.003) and Anosim (R = 0.5, *p* = 0.001). The runoff and stagnant samples were grouped into different groups.

**Figure 6:**
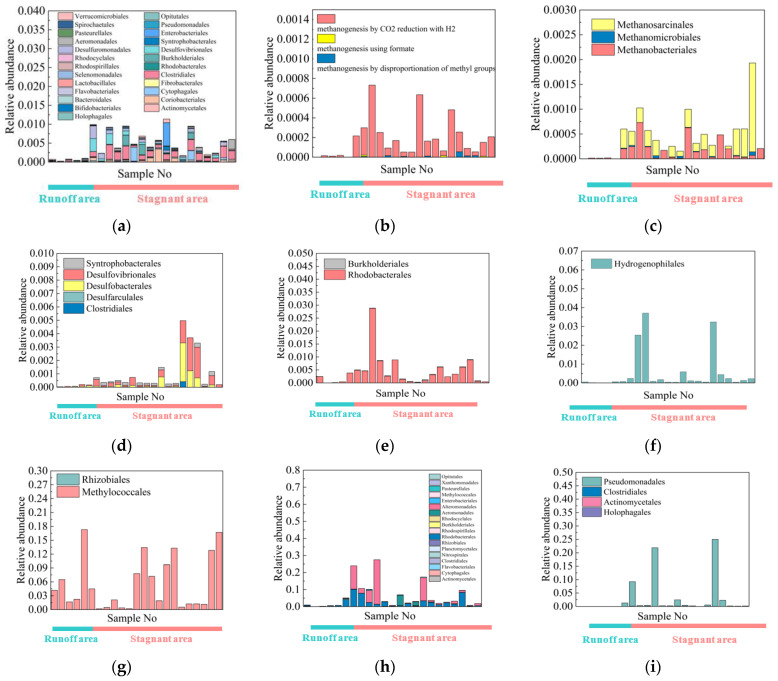
Histogram of relative abundance of C-N-S microorganisms in the study area: (**a**) fermentation bacteria; (**b**) different types of methanogens; (**c**) methanogens; (**d**) sulfate-reducing bacteria; (**e**) denitrifying bacteria; (**f**) dark sulfide oxidation bacteria; (**g**) methanotrophs; (**h**) aerobic chemoheterotrophic bacteria; (**i**) aromatic compound degradation.

The relative abundance of C-N-S functional communities increased in the stagnant zones (Figure S9 and Figure 6). This will be thoroughly discussed.

### 4.4. C-N-S-Cycling Species

In coals, there may be microbial CO_2_ reduction as well as methyl-type fermentation (acetoclastic) processes [23]. Both types of methanogenesis were found in our water samples, and the methanogenic system is dominated by CO_2_ reduction in the study area (Figure 6b). *Methanobacteriales*, *Methanosarcinales* and *Methanomicrobiales* were the main methanogen orders found in the research region (Figure 6c).

The methanogens found in the study were similar to those in other basins: *Methanobacteriales* was the main dominant order in the Konin Basin, Poland [24], and the coal seams in Japan [8]. In contrast to other coalbed methane basins, the research location had a distinct species of methanogens. The two primary methanogens in the water from the Alberta basin were *Methanosarcina* and *Methanobacteriales* [25]. The primary methanogens in the water from the Powder River Basin were *Methanolinea*, *Methanoregula*, *Methanospirillum*, *Methanolobus* and *Methanosaeta* [17]. The primary methanogens in the Ordos Basin were *Methanosaeta* [12].

Methane-oxidizing bacteria included *Rhizobiales* and *Methylococcales* (Figure 6g). They were also found in the Graissessac coal and Autun shale as methane consumers [26].

The biogeochemical cycle can be significantly impacted by the reactions of organic and inorganic sulfur compounds that make up sulfur metabolism in microorganisms [27,28,29,30,31]. Sulfate-reducing microorganisms included *Desulfovibrionales*, *Syntrophobacterales*, *Clostridiales*, *Desulfarculales* and *Desulfobacterales* (Figure 6d). Sulfate reduction is an anaerobic respiration process, similar to denitrification, which may be more potent in stagnant environments. *Clostridiales* are fermentative bacteria involved in coal biogasification, and they have been found in the Huaibei Coalfield [32], the Gippsland Basin [25] and western Canadian subsurface coalbeds [10]. Dark sulfide oxidation bacteria were mainly *Hydrogenphilales*.

We detected microorganisms related to the nitrogen cycle in the study area. *Nitrosomonadales* are the main AOB. *Nitrospirales* and *Nitrosomonadales* were involved in nitrification. Anammox bacteria contained members of the Candidatus Brocadiales group. *Rhodobacterales* and *Burkholderiales* were involved in denitrification (Figure 6e), and they were also found in the Graissessac coal [26]. Nitrogen fixation bacteria included *Methanomicrobiales*, *Clostridiales*, *Rhizobiales*, *Rhodospirillales*, *Burkholderiales* and *Rhodocyclales*.

Fermentation bacteria included *Holophagales*, *Actinomycetales*, *Bifidobacteriales*, *Coriobacteriales*, *Bacteroidales*, *Cytophagales* and *Flavobacteriales* (Figure 6a). *Actinomycetales* are involved in the synthesis of biosurfactants that aid in the solubilization and/or improvement of the bioavailability of hydrocarbons [33]. *Bacteroidales* and *Cytophagales* were discovered in a number of investigations of coal-associated enrichment cultures and were capable of fermenting hydrocarbons and aromatic chemicals [32].

Aromatic compound-degrading bacteria included *Holophagales*, *Clostridiales*, *Pseudomonadales* and *Actinomycetales* (Figure 6i). *Pseudomonadales* were the dominant strain. In the presence of nitrate, *Pseudomonadales* may degrade naphthalene anaerobically. *Pseudomonas* from coal seam water were able to produce rhamnolipid biosurfactants that could solubilize coal, showing a function in coal degradation [34].

Aerobic chemoheterotrophic bacteria were the dominant strain in the Shizhuangnan block, and they were mainly *Rhodobaterales*, *Alteromonadales* and *Aeromonadales*, suggesting their major role in the degradation of organic matter from the coal seams.

The relative abundances of the fermentation bacteria, methanogens, sulfate-reducing bacteria, nitrogen fixation bacteria, denitrifying bacteria, sulfide oxidation bacteria, aerobic chemoheterotrophic bacteria and aromatic compound degradation bacteria were higher in the stagnant than in the runoff areas (Figure 6). The bubble plot illustrates a more obvious change in the distribution features of C-N-S microorganism abundance in the study area, the abundance of all these C-N-S species increased significantly in the stagnant areas (Figures S9 and S13). However, methanotrophic and eutrophic bacteria and hydrocarbon-degrading bacteria seemed to be evenly distributed in the study area (Figure S9).

## 5. Discussion

### 5.1. Hydrochemical Mechanism in the Study Area

Due to the coal’s limited permeability in the southern Qinshui Basin, hydraulic fracturing is needed before CBM exploration. Water with an additional 2% KCl was usually used as fracture liquid and then drained out at the early pumping stage. Thus, it is important to evaluate the contribution of fracture liquid to the formation water. In this study, the drainage time of water sample collection and drainage wells is more than 8 years, so the influence of engineering factors can be eliminated.

As water and rock interact throughout the flow routes, the geochemical concentration of coal water is important for detecting hydraulic zones. The ions Na^+^, K^+^, Mg^2+^, Ca^2+^, Fe^3+^, CO_3_^2−^, Cl^−^, HCO_3_^−^, NO_3_^−^ and SO_4_^2−^ are often present in CBM-coproduced water. The pH of water ranged from 8 to 9. Na^+^ was the dominant cation. Most samples are enriched in HCO_3_^−^ and depleted in SO_4_^2−^.

The distribution characteristics of the ions from runoff to stagnant areas indicate that the hydrodynamics in the study area were mainly westward recharge flow. The fresh recharge water first accommodated the dissolution of salts and semiarid soils, such as gypsum, at shallow depths and was enriched in Ca^2+^ and Mg^2+^. In stagnant areas, anaerobic fermentation produces CO_2_ and forms HCO_3_^−^. HCO_3_^−^ will then cause the precipitation of dolomite and calcite, resulting in the depletion of Mg^2+^ and Ca^2+^. Indeed, Ca^2+^ and Mg^2+^ were constantly maintained at low concentrations in the stagnant areas, while Na^+^ increased with the distance from the recharge area.

The major source of Na^+^ and K^+^ in the coal is the dissolution of sylvite (KCl) or halite (NaCl). Furthermore, the weathering of silicate could discharge Na^+^ or K^+^. Na^+^ and K^+^ can be more abundant than Ca^2+^ or Mg^2+^ through cation exchange. The amount of mineralization (TDS) increased in the stagnated areas as a result of the water-rock reaction being more efficient. Cation exchange only occurs when the (Ca^2+^, Mg^2+^)/Na^+^ ratio is relatively high. It might be the main source of sodium close to the recharge area where the dissolution of Ca^−^ and Mg-bearing minerals is intensive. However, in stagnant areas, Na^+^ becomes the dominant cation, and cation exchange declines. The other possibility is the dissolution of sodic silicate. This would enhance the concentrations of both Na^+^ and HCO_3_^−^.

The concentrations of SO_4_^2−^ and H^+^ in coal water may increase due to the oxidation of pyrite close to surface recharge zones. Nitrogen-fixing bacteria on the surface of the soil can convert N_2_ into bioavailable nitrogen. AOB and NOB further oxidize NH_3_ to NO_3_^−^. As a result, atmospheric precipitation carries these ions from the recharge zone to the deep reduction zone. Moreover, microbial degradation of amino acids will produce NH_4_^+^.

SO_4_^2−^ reduction, denitrification, NO_3_^−^ reduction and Fe^3+^ reduction are significant anaerobic oxidation pathways that significantly affect the S and C cycles. The concentrations of NO_3_^−^, SO_4_^2−^ and Fe^3+^ were more abundant in the runoff recharge zone and decreased from the east (runoff areas) to the west (stagnant areas). These three ions are electron acceptors of anaerobic respiration microorganisms. The concentrations of Fe^3+^, NO_3_^−^ and SO_4_^2−^ were low in the study area, especially in the stagnant areas, showing the utilization of these electron receptors by microbial anaerobic respiration. When these ions are consumed excessively, anaerobic fermentation microorganisms would provide substrates for methanogens, such as H_2_, CO_2_ and acetic acid. 

DIC is a measure of the total amount of inorganic carbon in a solution, including bicarbonate, carbon dioxide, carbonic acid and carbonate. Carbonate dissolution and organic decomposition are the two primary DIC contributors in groundwater. While δ^13^C_DIC_ is often negative in natural water systems, it is frequently positive in coproduced water from CBM because of carbon isotope fractionation by methanogenesis, ranging from 10% to 30%. As methanogens prefer to use ^12^C, which leads to the enrichment of δ^13^C_DIC_, the values of δ^13^C_DIC_ were more positive in the stagnant areas than in the runoff areas, demonstrating that methanogenesis was more active in the stagnant areas. Indeed, we detected a higher abundance of methanogens in the stagnant areas.

The source of the groundwater could be determined using oxygen and hydrogen isotopes. The following equation for the local meteoric water line was used: δD = 7.9δ^18^O + 8.2. Figure 3a shows that the distribution of the water samples taken was close to the atmospheric precipitation line, indicating that atmospheric precipitation was primarily the source of coal seam water. The isotopes are scattered above the LMWL due to D drift, and others are below or to the right due to ^18^O drift. The ^18^O enrichment in formation water could be a result of mixing with brine water, evaporation and fluid–rock interactions under high temperatures [22]. Jia suggested an evaporation line (EL) in Taiyuan city (~250 km north of the study site) based on surface water data: δD = 5.744 × δ^18^O − 28.285. Our data are located between the LMWL and EL. This result demonstrates the different sources of meteoric water in the coal seams, which is mainly a mixing of fresh meteoric water with strongly evaporated meteoric water.

### 5.2. Metagenome Sequencing Results Verified the C-N-S Microbial Differences between the Stagnant Areas and Runoff Areas

We used metagenome results to compare the distribution of the abundance of functional genes between the runoff and stagnant areas, including methane metabolism genes, polysaccharide degradation genes, aromatic compound degradation genes, N-cycling genes and S-cycling genes.

Numerous studies have been conducted on methanogens’ functioning mechanism [35,36,37]. The abundance of methanogenesis metabolism genes, including *mcrG, mcrB,* and *mcrA,* the essential enzymes in methanogenesis, rose in the stagnant zones (Figure S10a). Additionally, the hydrogenotrophic methanogenesis genes *mer*, *mtd*, *FwdA*-*FwdH*, *mtrA*-*mtrH* and *MvhADG*–*HdrABC* were more abundant in stagnant areas. Similarly, in stagnant areas, acetoclastic methanogenesis genes *Codh*–*Acs* and methylotrophic methanogenesis genes *MtaA*-*MtaC* were also more prevalent. Additionally, the abundances of *cofC*, *cofG*, *cofD*, *fbiC*, *cofH* and *cofE*, which are associated with F_420_ biosynthesis; *mfnE*, *mfnB*, *mfnD* and *mfnF*, which are related to methanofuran biosynthesis; and *comC*, *comD* and *comE,* which are related to CoM biosynthesis, were greater in the stagnant zones. This shows that the western stagnant areas show an increase in all forms of methanogens.

In the stagnant samples, the abundance of the N_2_-fixing genes *nifK*, *nifD* and *nifH* was higher. In addition, nitrification and denitrification processes increased in the stagnant areas, as indicated by increased *nirK*, *nosZ*, *norC*, *napA*, *napB*, *nrfA*,and *amoB* genes (Figure S10a). Higher nitrite/nitrate concentrations would result from an increase in the nitrification and N_2_-fixing genes, which would also result in more abundant genes for different forms of reductive respiration that use NO_3_^−^/NO_2_^−^ as electron acceptors, such as *nrfA* used for dissimilatory NO_3_^−^ reduction; *nirK*, *norC* and *nosZ* for denitrification; *narG*, *napA*, *napB, narH* and *narI* for dissimilatory NO_3_^−^ reduction/denitrification; and *nirA* used for assimilatory NO_3_^−^ reduction (Figure S10a).

Reactions of sulfur substrates make up the sulfur metabolism in microorganisms, which can be a significant contributor to the biogeochemical cycle [27,28,29,30,31]. Sulfate reduction, a kind of anaerobic respiration, is similar to denitrification and may be more effective in stagnant environments. According to Welch’s *t*-test, the sulfur metabolic genes were more abundant in the stationary regions than in the runoff areas (Figure S10a), suggesting that the stagnant areas had stronger microbial S cycling. These genes included *dsrAB*, which encodes dissimilatory sulfite reductase; *sat*, which encodes sulfate adenylyltransferase; *sir*, which encodes sulfate reductase; *cysN*, which encodes sulfate adenylyltransferase; and *aprAB*, which encodes adenylylsulf. This is consistent with the findings of our geochemical tests, which show that sulfate reduction was higher in stagnant areas.

Organic carbon from surface plants will seep into the coal seam with atmospheric precipitation. Numerous microbial functional groups crucial for C breakdown were altered by changes in hydrodynamic circumstances (Figure S10b). The carbon-cycling genes were involved in the degradation of complex carbon compounds, such as starch, hemicellulose (xylanase), cellulose (cellulase), chitin (chitinase), starch (1,4-alpha-glucan branching enzyme) and lignin (ligninase).

The genes involved in the breakdown of labile/recalcitrant C (cellulose, chitin, hemicellulose, lactose, lignin, pectin, starch), including glucose-6-phosphate isomerase, chitinase, mannose 6-dehydrogenase, beta-N-acetylhexosaminidase and N-acetylglucosamine kinase, were more abundant relative to runoff area samples in stationary regions. These genes degrade the associated plant organic carbon and include those involved in degrading chitin (for example, beta-hexosaminidase), cutin (for example, cutinase), lignin (for example, ligninase, manganese peroxidase and laccase), pectin (for example, endopolygalacturonase, pectate lyase, pectinesterase and carbohydrate esterase) and starch (for example, α-amylase and cyclomaltodextrinase).

Increases in the genes responsible for polysaccharide breakdown showed that surface plant carbon in the stagnant regions may be degraded. Once polysaccharides were degraded into monosaccharides, the bacteria in the coal metabolized the alternative carbon sources (mannose, glucose, xylose, galactose, and arabinose) via the Entner–Doudoroff pathway and pentose phosphate pathway, indicating a high affinity to the monosaccharides obtained from plants. Formic acid, methanol, CO_2_ and acetic acid were formed, which may be used by hydrogenotrophic and acetoclastic methanogens as acceptable precursors.

Hydrocarbons, molecules that contain only carbon and hydrogen atoms, are widely used by microbes as electron donors [38,39]. Aromatic hydrocarbons are important substrates for microbial degradation in coal. They can be degraded both aerobically and anaerobically (by adding fumarate). In coal and shale formation water, aromatic chemicals were found [38]. Aromatic compounds could also be biodegraded in the San Juan Basin, Powder River and lignite samples collected in the Konin region [34]. Genes involved with aromatic compound degradation were more abundant in stagnant areas than that in runoff samples (Figure S10c). These genes included alkane degradation genes such as enoyl-CoA hydratase and ethylbenzene dioxygenase; benzoate degradation gene *benB-xylY*; biphenyl degradation genes such as *bphA*, *bphB*, *bphC* and *bphD*; naphthalene degradation genes such as *nahAa*, *nagAc*, *nahD* and *nahE*; and PAH degradation genes such as catechol 2,3-dioxygenase and muconate cycloisomerase.

The heavy hydrocarbons in the study area may have been almost cracked, the remaining alkane could be catalyzed by a monooxygenase, and the end product of the reaction sequence is a fatty acid equal in length to the initial hydrocarbon. The fatty acid is subsequently subjected to beta-oxidation, in which two of its carbon atoms are removed from the fatty acid at a time. Acetyl-CoA and a new fatty acid with two fewer carbon atoms than the original fatty acid are released after one beta-oxidation cycle. The acetyl-CoA formed by β-oxidation is used by the citric acid cycle to produce acetic acid, an important substrate for methanogenesis.

Microorganisms may utilize aromatic (ringed) hydrocarbons as electron donors both aerobically and anaerobically. The synthesis of catechol or a structurally comparable chemical via catalysis by oxygenase enzymes often occurs as the early stage of the metabolism of these compounds, some of which include multiple rings, such as naphthalene or biphenyls. Once catechol is produced, it can be broken down further to produce substances such as acetyl-CoA, succinate and pyruvate that can enter the citric acid cycle. They were used as substrates for methanogens in coal seams.

### 5.3. Major Environmental Attributes Shaping Microbial Community Functional Structure

Environmental factors, including temperature, oxygen, pH and redox conditions, control the distribution characteristics of microorganisms, especially microorganisms related to C-N-S cycling, including anaerobic respiratory bacteria and fermentation bacteria [40,41]. Hydrogeological conditions are a crucial controlling factor for CBM enrichment [42,43]. The synclines west of the Shizhuangnan block weakened the coal’s hydrodynamic strength, and the study area’s redox environment changed significantly from the oxic runoff recharge zone of the basin edge to the stagnant areas [22].

**Figure 7:**
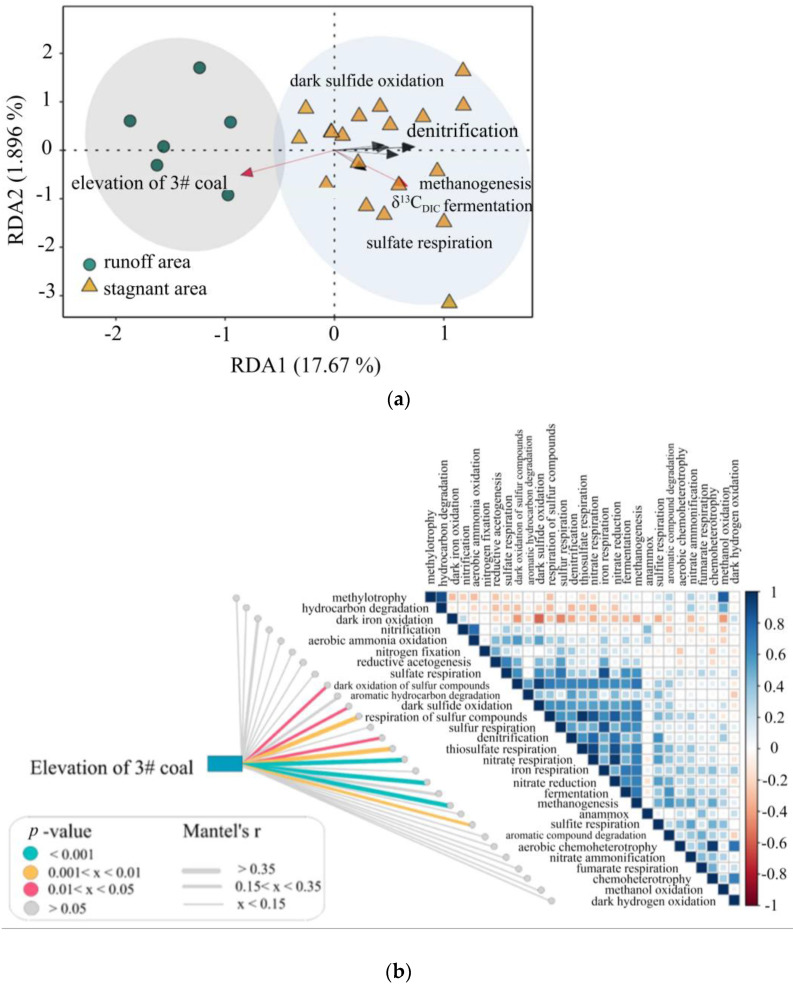
(**a**) Linkages between microbial community and environmental variables. RDA analysis displays microbial community (symbols, including C-N-S microorganisms such as fermentation bacteria, methanogens, sulfate-reducing bacteria, denitrifying bacteria and dark sulfide oxidation bacteria) and environmental variables (arrows, including elevation of #3 coal). The value of the axis is the variance percentage explained for the axis. (**b**) Pairwise comparisons of functional communities are shown with a color gradient denoting Spearman’s correlation coefficient. Taxonomic and functional community structures were related to each environmental factor by Mantel tests. Edge width corresponds to the Mantel’s r statistic for the corresponding distance correlations, and edge color denotes the statistical significance.

Different hydraulic zones in the Shizhuangnan block exhibit substantial differences in environmental conditions [22]. Various microbiomes in distinct hydrological areas have different geochemical characteristics, and hydrological circumstances have a significant regulating impact on C-N-S microorganisms. This paper hypothesizes that the functional composition of microbes changes from runoff zones to stagnant zones. This view was verified by the RDA and Mantel test, showing that weakened hydrodynamics could greatly stimulate the C-N-S microorganism distribution (Figure 7). The elevation of the #3 coal reflects the change in hydrological conditions, and most of the C-N-S microorganisms had strong correlations with the elevation of the #3 coal, showing that these microorganisms are important in controlling C-N-S cycling.

### 5.4. Degradation Mechanism of Organic Matter and Biogenic Methane 

**Figure 8:**
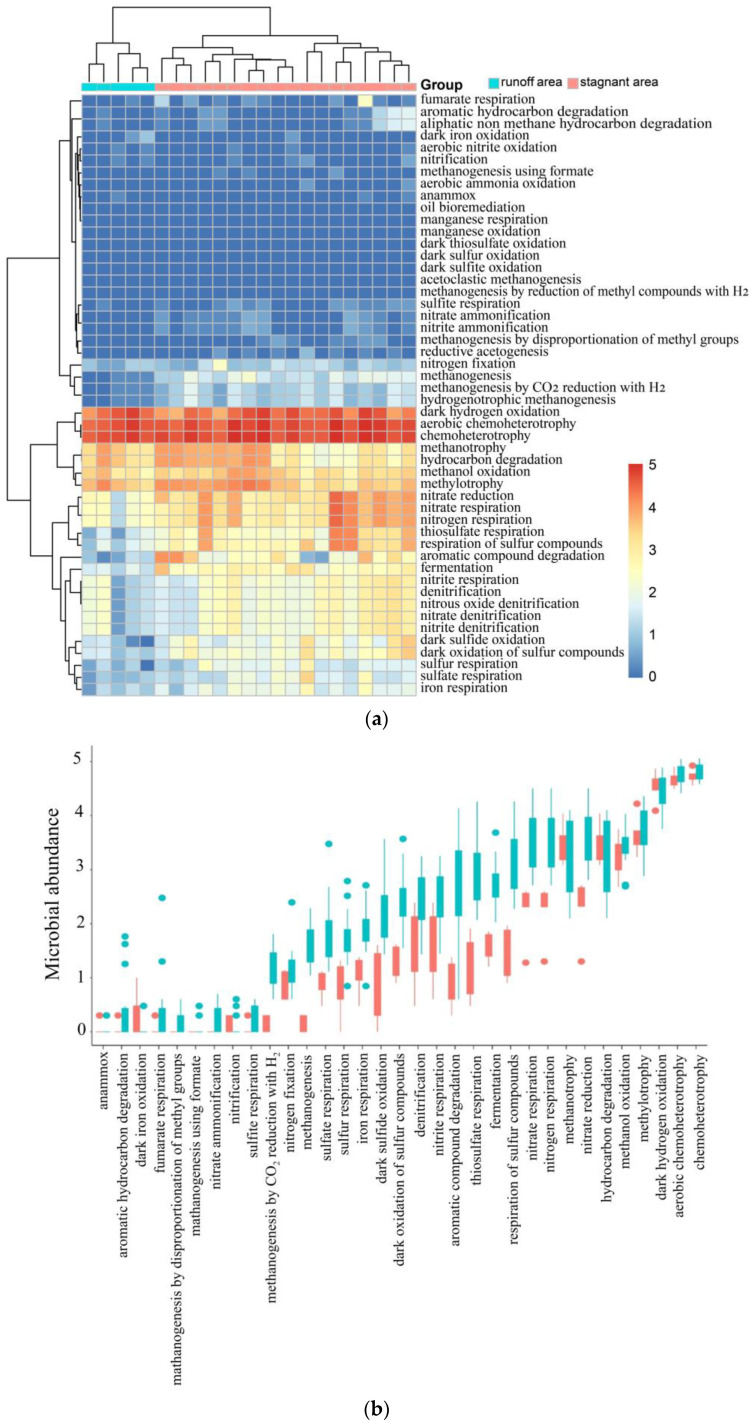

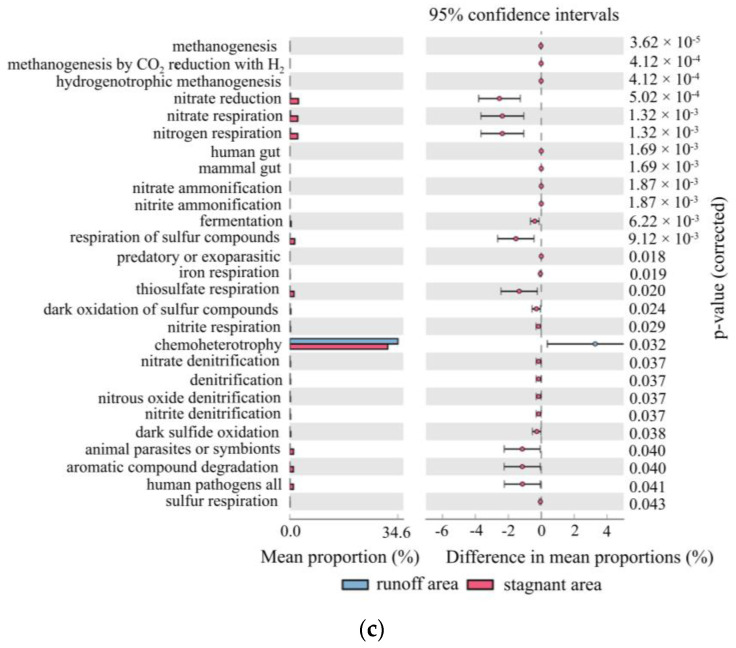
(**a**) Heatmap showing relative abundance of C-N-S microorganisms of each sample in runoff and stagnant areas. The relative abundance of microorganisms was converted into a log_10_ (relative abundance × 10^6^) scale for better exhibition in the heatmap. (**b**) Comparison of C-N-S microorganisms between runoff and stagnant areas. The relative abundance of genes was converted into a log_10_ (relative abundance × 10^6^) scale for better exhibition. (**c**) Analysis of different relative abundances of C-N-S microorganisms between runoff and stagnant areas using Welch’s *t*-test with FDR correction in STAMP (95% confidence intervals, *p* < 0.05).

By classifying the flora in the study area according to the C-N-S function, the heatmap shows that the C-cycling microbial communities, including methanogenesis, fermentation, and aromatic compound degradation communities, were enriched in the stagnant areas (Figure 8a,b and Figure S13). The significant difference between stagnant areas and runoff areas was verified by Welch’s *t*-test (Figure 8c) (*p* < 0.05, 95% confidence intervals).

Atmospheric precipitation carries organic matter, such as plant labile C (cellulose, hemicellulose, etc.) and recalcitrant C (chitin, aromatics, etc.), and penetrates the deep coal from the surface outcrop of the coal seams. In the strong reduction zone of deep coal seams, C-N-S microorganisms will degrade this organic matter and produce H_2_, CO_2_ and CH_3_COOH through fermentation under appropriate temperature and pressure. Methanogens can use these substrates and convert CO_2_ into biogenic CH_4_ to realize the recycling of energy. In medium- and low-rank coal seams, microorganisms also degrade the organic matter in coal seams.

Vegetation C serves as a significant substrate for microbial decomposition in plateau frozen soil, permafrost, forest, grassland and residue waste degradation [44,45,46,47,48,49]. This investigation supported the hypothesis that vegetation C originated from plants on the ground. In the research region, the coal temperature in the stagnant areas rose relative to the runoff areas, which would be more advantageous for the degradation of both the labile C and the resistant C. Microorganisms would benefit from the surface’s organic carbon breakdown, and the monosaccharides that were formed may have also offered substrates for carbohydrate metabolism [19,50,51]. Metagenome resequencing in the research region identified genes related to L-fructose utilization, carbohydrate hydrolases, galactose and lactose utilization, mannose metabolism and xylose utilization.

In addition to organic carbon from the surface, coal authigenic organic matter could also be used as a substrate for microorganisms [52].

Alkanes, toluene, alkylbenzenes and PAHs are typical coal monomers that can be degraded aerobically or anaerobically by aromatic compound degradation microorganisms. The related functional genes were detected in metagenome sequencing and enriched in stagnant areas. Hexadecanoic acid, n-alkanes, hopanes, terpanoids and other saturated hydrocarbons may be produced from coal extracts. Metabolites such as fatty acids may be present in coal fluids because of hydrocarbon biodegradation. Methylalkylsuccinates are typical byproducts of n-alkane biodegradation by adding fumarate. These intermediates can undergo further oxidation to become substrates for methanogenesis. Coal formation water is frequently contaminated with aromatic chemicals. Researchers have also found PAHs such as biphenyls.

Because the degradation of polysaccharides and aromatic hydrocarbons provides substrates for fermentation, these substrates are further fermented into substrates for methanogens, so methanogenesis is stronger in the stagnant zone [53,54,55,56].

Anaerobic methane-oxidizing microorganisms (*M. oxyfera* and *ANME*) were not detected in the water samples, and their absence facilitated the preservation of secondary biogenic coalbed methane, especially in the stagnant areas. As the outcrop of elevated coal obtained a meteoric supply of organic C, methanogens started to proliferate, so the study area is currently in the secondary biogenic gas generation stage. 

### 5.5. N Cycling: The Mechanism of N Cycling in Microorganisms

N cycling in soils, permafrost and marine oxygen-minimum zones has been well studied [57,58,59,60]. All microorganisms require some form of nitrogen, such as nitrate, nitrite or ammonium, for the synthesis of essential biological components such as amino acids and nucleotides [61,62,63,64]. Various types of N-cycling communities were detected in the study area, including nitrogen fixation, AOB, nitrification, dissimilatory/assimilatory NO_3_^−^ reduction, denitrification, and synthesis of amino acids and nucleotides. Most (56%) N-cycling microorganisms increased in the stagnant areas (Figure 6 and Figure 7). 

The N-cycling microbes and their associated genes may improve the availability of nutrients (particularly N) in stagnant coal reservoirs since N is a constraint for microorganisms in groundwater, which would be significant for C dynamics [65,66]. As a result of the increased N intake, C metabolism, including the metabolism of cellulose and mannose and methanogenesis, which increased in response to hydrodynamic circumstances, may be promoted. This is consistent with recent studies on how microbes react to warming effects in frozen soil [67,68].

### 5.6. Relationship of C-N-S Functional Microorganisms

As demonstrated in the heatmap in Figure 7a, C-N-S microorganisms were often abundant in the stagnant zones, suggesting that they were related to each other. The increase in N-cycling microorganisms may improve the availability of nutrients in coal seams, which may then impact C-cycling metabolism. Carbon metabolisms were related too. The principal source of C for microbes is the breaking of polysaccharides into simple sugars [19,50,51]. Degradation genes for simple sugars were prevalent in the metagenome sequencing data (Figure S10), including L-fructose, mannose, lactose, galactose and xylose metabolism [69]. In addition, the alkane and aromatic hydrocarbons in coal could also provide substrates for methanogens.

Nitrate and sulfate were limited in anaerobic coal reserves, particularly in stagnant areas. Fermentation is a crucial mechanism for the breakdown of monosaccharides and provides substrates for methanogens. Acetate, propionate, lactate, and ethanol were produced together with H_2_ and CO_2_ during fermentation. These fermentation genes were prevalent in the stagnant areas, indicating higher synthesis of these metabolic substrates. Methanogens were able to utilize H_2_ and acetic acid, and they were more abundant in stagnant areas.

We predict that the oxygen carried from the surface would be gradually consumed as the groundwater flows through the deep stagnant areas. In the stagnant areas, anaerobic respiration and microbial anaerobic fermentation gradually strengthened, consuming Fe^3+^, NO_3_^−^ and SO_4_^2−^ and resulting in the reduction in these ion concentrations in the stagnant areas (Figure 5c–e). We detected that nitrate respiration, sulfate respiration and iron respiration bacteria were more enriched in the stagnant areas, which verifies the above view, suggesting enhanced C-N-S cycling in the stagnant areas.

**Figure 9:**
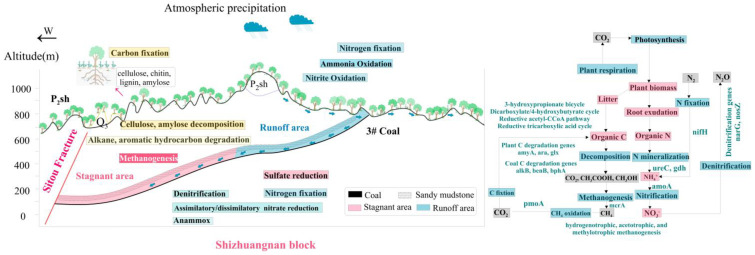
Microbial C-N-S cycle pattern in Shizhuangnan Block.

The microbiological C-N-S pattern in the high-production stagnant areas was constructed according to the studies mentioned above. The degradation and fermentation of terrestrial organic matter and coal primary organic matter were more enriched in the stagnant areas, providing a substrate for methanogens. Therefore, the study area has a stronger methanogenic effect in the stagnant areas, with a stronger potential for secondary biogas generation, as shown in Figure 9.

### 5.7. Machine Learning Classification of C-N-S Microorganisms 

**Figure 10:**
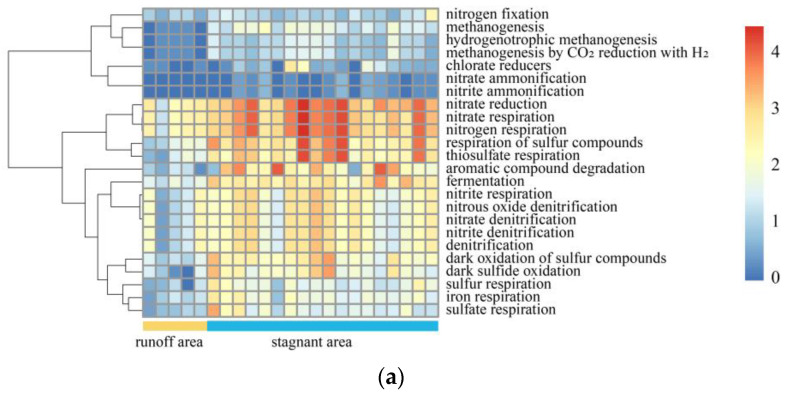

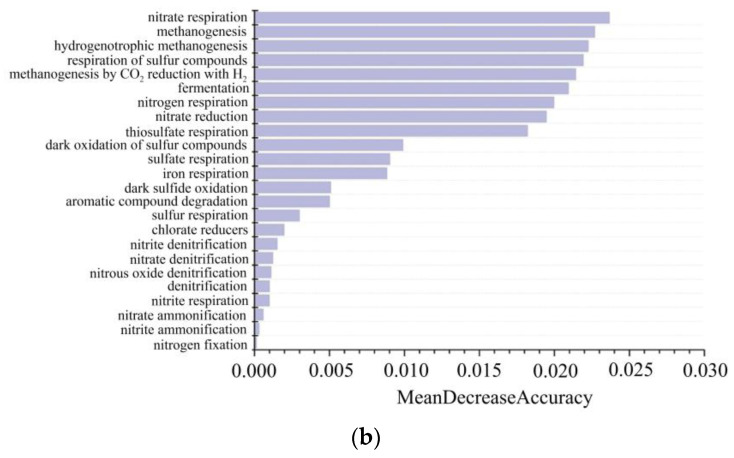
(**a**) Heatmap showing microorganisms’ relative abundance that changed significantly between runoff and stagnant areas; these microorganisms were chosen by machine learning calculation. The microorganisms’ relative abundance was converted to log_10_ (relative abundance × 10^6^) for better exhibition in the heatmap. (**b**) Importance ranking of MeanDreaseAccuracy calculated by randomForest packages.

C-N-S microbes could be used to distinguish the hydraulic areas in a random forest model because they varied considerably between the two areas (Figure 10a).

These most important community functions included methanogenesis, nitrate respiration, fermentation, nitrate reduction, dark oxidation of sulfur compounds, sulfate respiration, iron respiration, chlorate reduction, aromatic compound degradation, denitrification, ammonification and nitrogen fixation.

The relative abundances of the functional microorganisms chosen by the random forest model are shown in Figure 10b. Their abundance exhibited a sensitive change between different hydraulic areas, and this response may be employed as an efficient gauge to distinguish them. The accuracy of our model in the research region was checked using the 16S sequencing results from 2016; it revealed an accuracy of 90%. 

### 5.8. Indication of Microbial Sequencing in the Process of Coalbed Methane Exploration and Development

This study found biogenic methanogenesis in high-ranking coal reservoirs and analyzed the sources of substrates required for methanogenesis, including surface plant organic carbon and in situ organic matter in coal seams, and the stronger degradation of organic matter and biogenic methanogenesis in the stagnant zone. 

Our study shows that methanogenic archaea are sensitive to the hydrodynamic and water chemical fields of coal seams, and the hydrological stagnation zone with strong reduction is usually favorable to the preservation of coalbed methane, which is significant for guiding the exploration and development of coalbed methane. In this study, a small number of drainage wells in the study area are located in the hydrological runoff zone with low gas content and gas production (Figure S11), which reduces the benefits of coalbed methane development. 

Although the geochemical characteristics of the produced water of CBM extraction wells, such as water ion concentration, can be used as indicators to identify hydrodynamic conditions, the water ion concentration is more closely related to minerals in coal and environmental factors. Microbial sequencing has the advantages of low cost and strong indication. This study combines geochemical data with microbial sequencing data to provide a more reliable basis for identifying the high-CBM-production area (stagnant zone), which is in full agreement with the current production capacity of the drainage wells and can be used as an effective basis for exploration and development of other development blocks in the Qinshui Basin. The prediction analysis of gas and water production in the runoff and stagnant areas shows that the gas production of the drainage wells in the stagnant areas is high, and the stable production period lasts for 11 years, with gas production being maintained at 1000–3000 m^3^/day and a high potential for increasing production; the stable production period of the well group in the runoff areas is 4 years, with a gas production of 500–1000 m^3^/day and limited potential for increasing production (Figure S12).

## 6. Conclusions

In conclusion, runoff areas and stagnant areas may be distinguished within the research area’s hydrodynamic zone. They each had unique hydrochemical and hydrodynamic properties. Compared to runoff areas, the stagnant areas showed increased TDS, reservoir pressure and gas concentration. The concentrations of Fe^3+^, SO_4_^2−^ and NO_3_^−^ decreased from runoff areas to stagnant areas in the west, demonstrating that they were used more efficiently in stagnant areas.

C-N-S-cycling microorganisms, including those engaged in methanogenesis, nitrate respiration, fermentation, nitrate reduction, dark oxidation of sulfur compounds, sulfate respiration, iron respiration, chlorate reduction, aromatic compound degradation, denitrification, ammonification and nitrogen fixation, were more abundant in the stagnant areas.

The increase in biogenic methane and decrease in Fe^3+^, SO_4_^2−^ and NO_3_^−^ in the stagnant areas were caused by an increase in the number of microorganisms involved in C-N-S cycling.

In the stagnant areas, there were larger concentrations of genes involved in anaerobic respiration activities, including N-cycling genes, methanogenesis genes and S-cycling genes. The C-N-S microorganisms had a sensitive response to the hydrogeological conditions, and this response was an efficient factor distinguishing the runoff areas and stagnant areas. The C-N-S microorganisms might be utilized as indicators based on machine learning models to forecast high-yield-potential areas, as the high-producing wells are all located in the western stagnant zone.

## Data Availability

The raw metagenome sequencing data generated in this study have been deposited in the NCBI database under the accession codes PRJNA925286 and PRJNA925297.

## Supplementary Materials

The following supporting information can be downloaded at: https://www.mdpi.com/article/10.3390/microorganisms11020497/s1.
