# Supplementary material for: Microbiome of High-Rank Coal Reservoirs in the High-Production Areas of the Southern Qinshui Basin
Source: Microorganisms. 2023 Feb 16;11(2):497. doi: 10.3390/microorganisms11020497 (PMC9963281; doi:10.3390/microorganisms11020497)
Supplement: Supplementary file 1 [file microorganisms-11-00497-s001.zip › microorganisms-2159817-supplementary.pdf]

microorganisms

MDPI

Article

Microbiome of High-Rank Coal Reservoirs in the High-Production Areas of the Southern Qinshui Basin

Wei Shi [1,2,3] and Shuheng Tang [1,2,3,*] and Songhang Zhang [1,2,3]

1 MOE Key Lab of Marine Reservoir Evolution and Hydrocarbon Enrichment Mechanism, Beijing 100083, China
2 MOLR Key Lab of Shale Gas Resources Survey and Strategic Evaluation, Beijing 100083, China
3 School of Energy Resources, China University of Geosciences (Beijing), Beijing 100083, China
* Correspondence: tangsh@cugb.edu.cn

Supplementary Figures

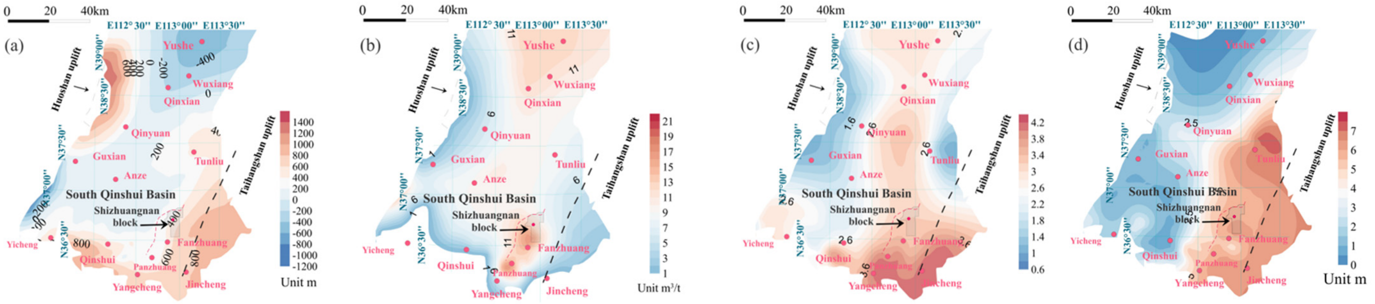

Figure S1. Contour map of the #3 coal-seam reservoir characteristics: (a) Contour map of the #3 coal-seam reservoir elevation (m); (b) Contour map of the #3 coal-seam reservoir gas content (m³/t); (c) Contour map of the #3 coal-seam reservoir vitrinite reflectance; and (d) Contour map of the #3 coal-seam reservoir thickness (m).

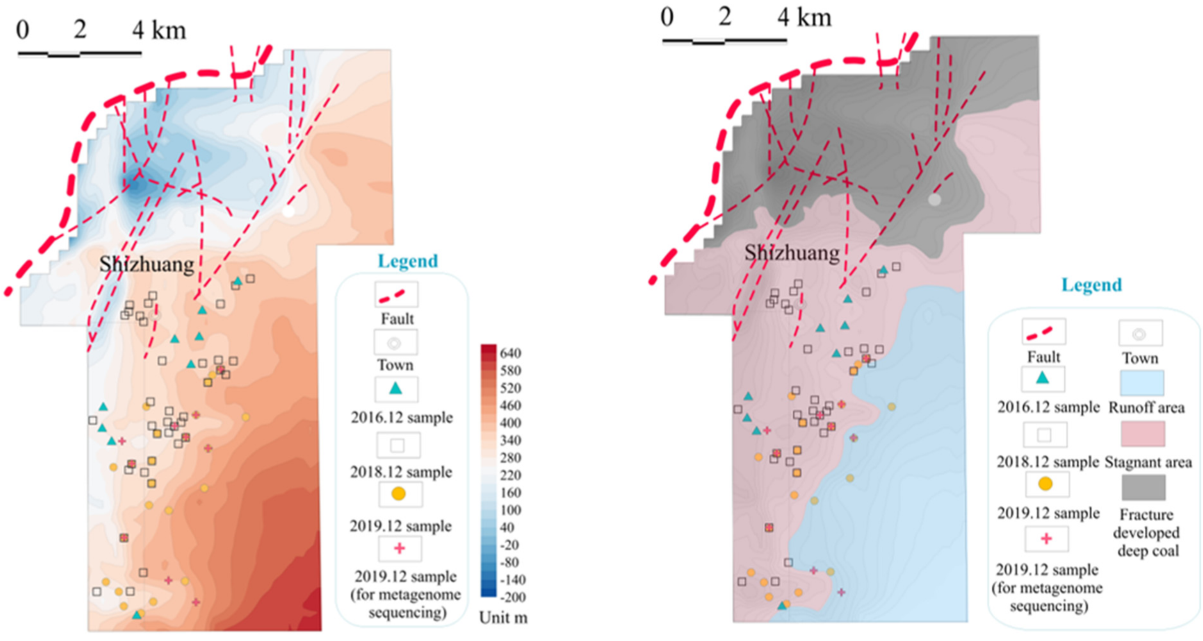

(a) (b)

Figure S2. (a) Sample distribution in the study area. The sampling points with different colors and shapes represent three geochemical and microbiome tests in the study area from 2016 to 2019, as shown in the legend. The base map is the elevation contour of #3 coal seam (m); (b) Division between the runoff and stagnant areas. Different colors represent different hydrological zones, including runoff areas in the recharge zone, stagnant areas and deep coal areas, as shown in the legend.

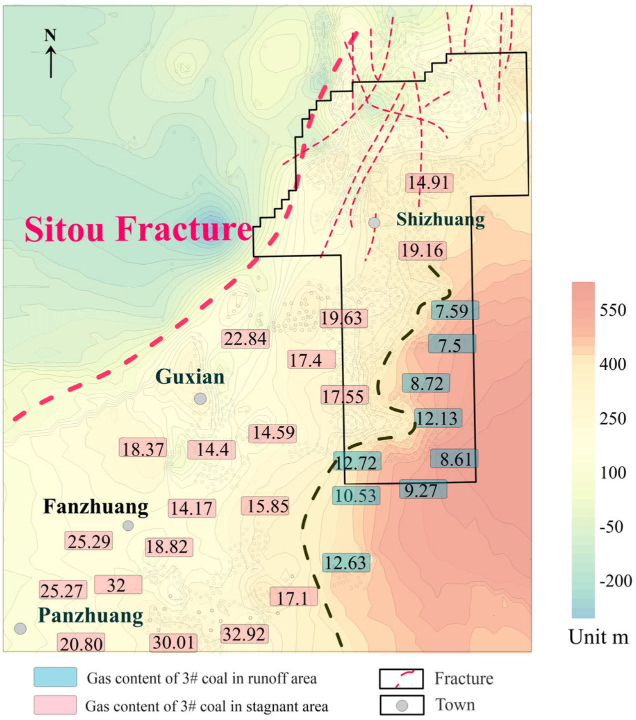

Figure S3. #3 coal-seam reservoir gas content (m^3/t) in the study area. The base map is a contour map of the #3 coal-seam reservoir elevation (m). The red dotted line represents the Sitou fracture, and the black dotted line represents the boundary line between the runoff and stagnant areas.

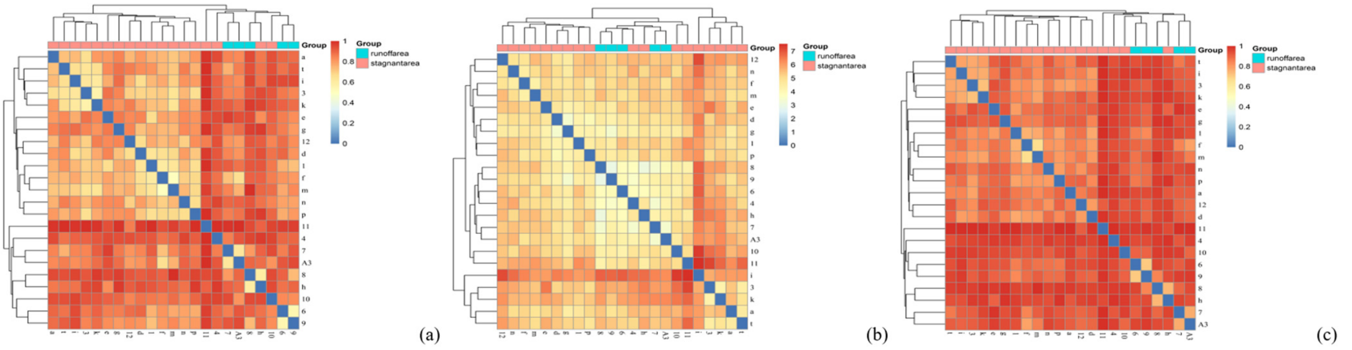

Figure S4. β Diversity heat map based on the microbe's abundance. (a) Bray Curtis distance; (b) Euclidean distance; (c) Jaccard distance. The three distance matrices showed that the runoff samples had more similar microbial composition.

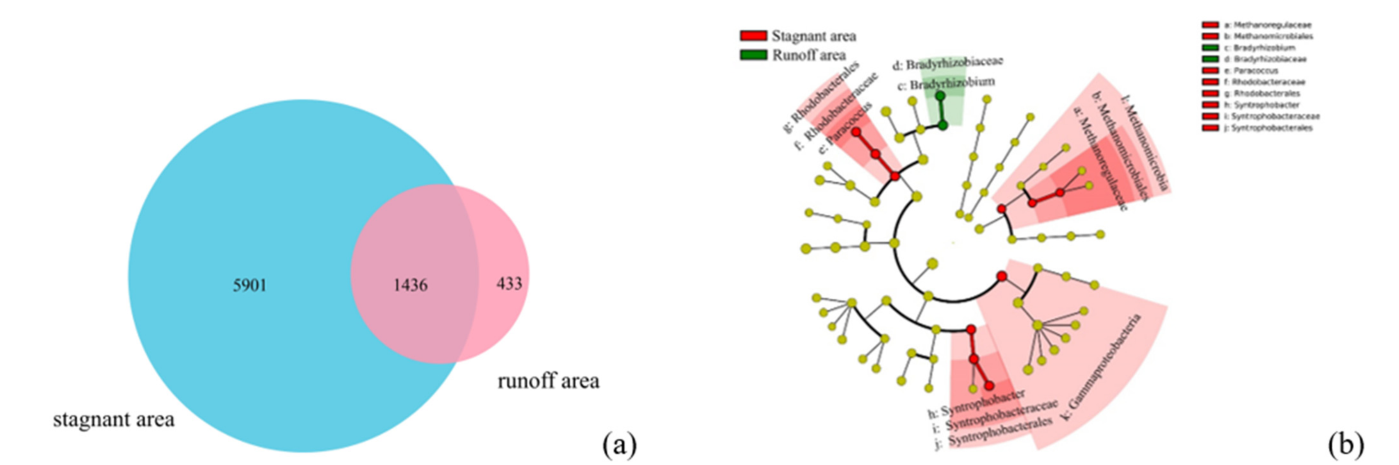

Figure S5. (a) Venn diagram of microbial species in runoff area sand stagnant areas. (b) Comparison between stagnant areas (red) and runoff areas (green) bacterial and archaeal community composition shown with a phylogenetic cladogram created using LEfSe analysis.

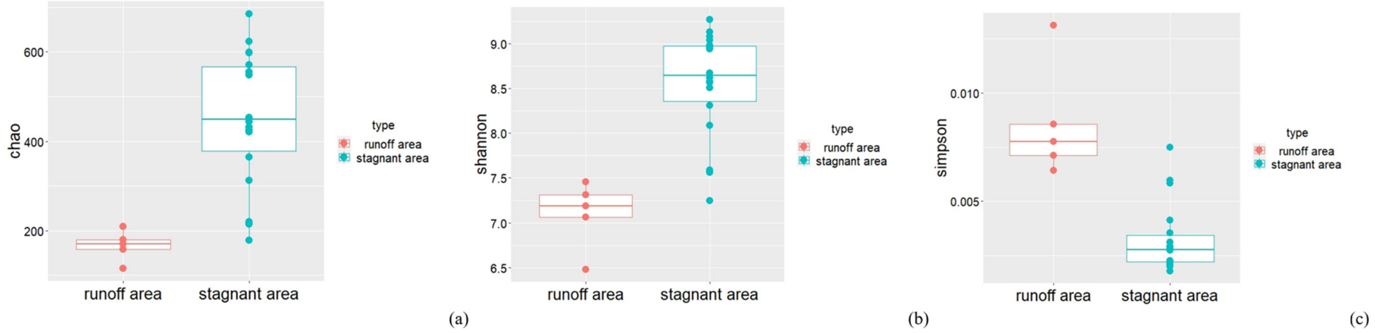

Figure S6. Comparison of alpha index of the microbiota between runoff areas and stagnant areas. (a) Chao index; (b) Simpson index; (c) Shannon index. The horizontal bars within boxes represent medians. Data in two areas show the consistent trend that the microbiota of stagnant areas show higher alpha diversity than those of runoff areas.

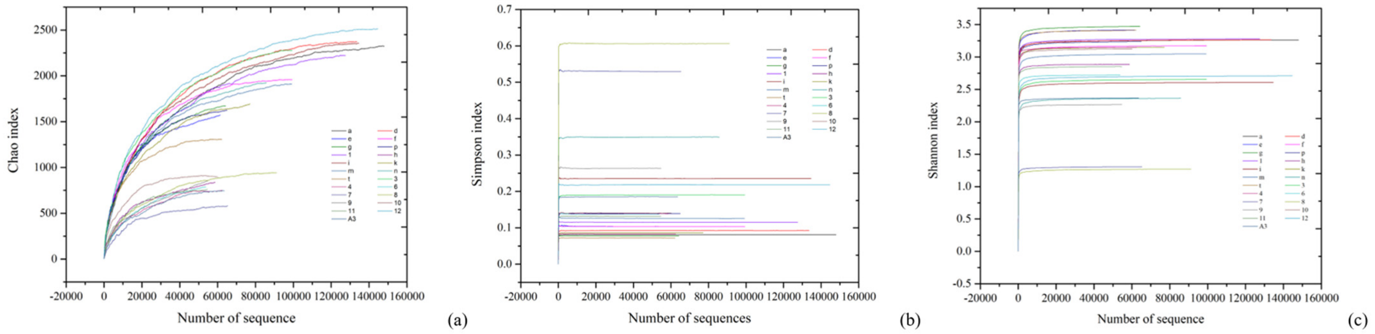

Figure S7. Alpha Rarefaction curves. The ordinate is the corresponding alpha index, and each curve is a sample. (a) Chao index; (b) Simpson index; (c) Shannon index. Rarefaction curves of alpha index of the water microbiota reach the saturation stage with increasing sequencing depth, indicating that the microbiota in our test capture most bacteria members from each sample.

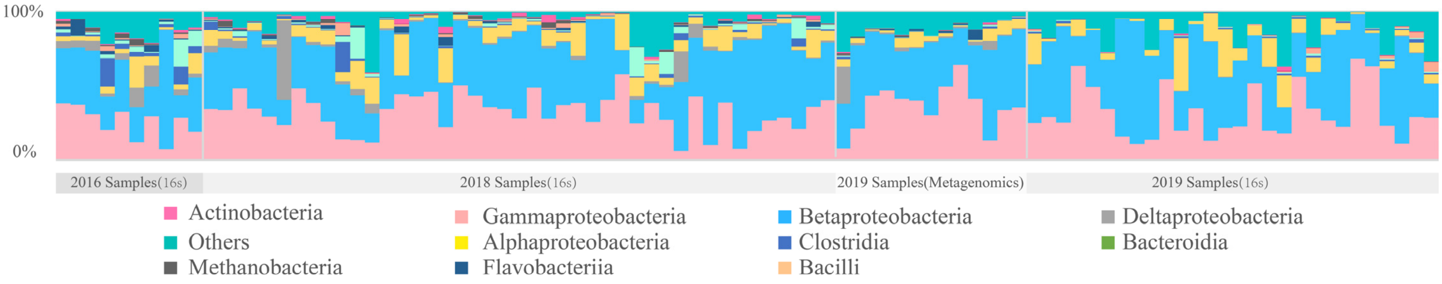

Figure S8. Species distribution map in the study area. Samples were collected in the study area from 2016 to 2019. 16s rRNA sequencing and metagenomic sequencing were used to determine species.

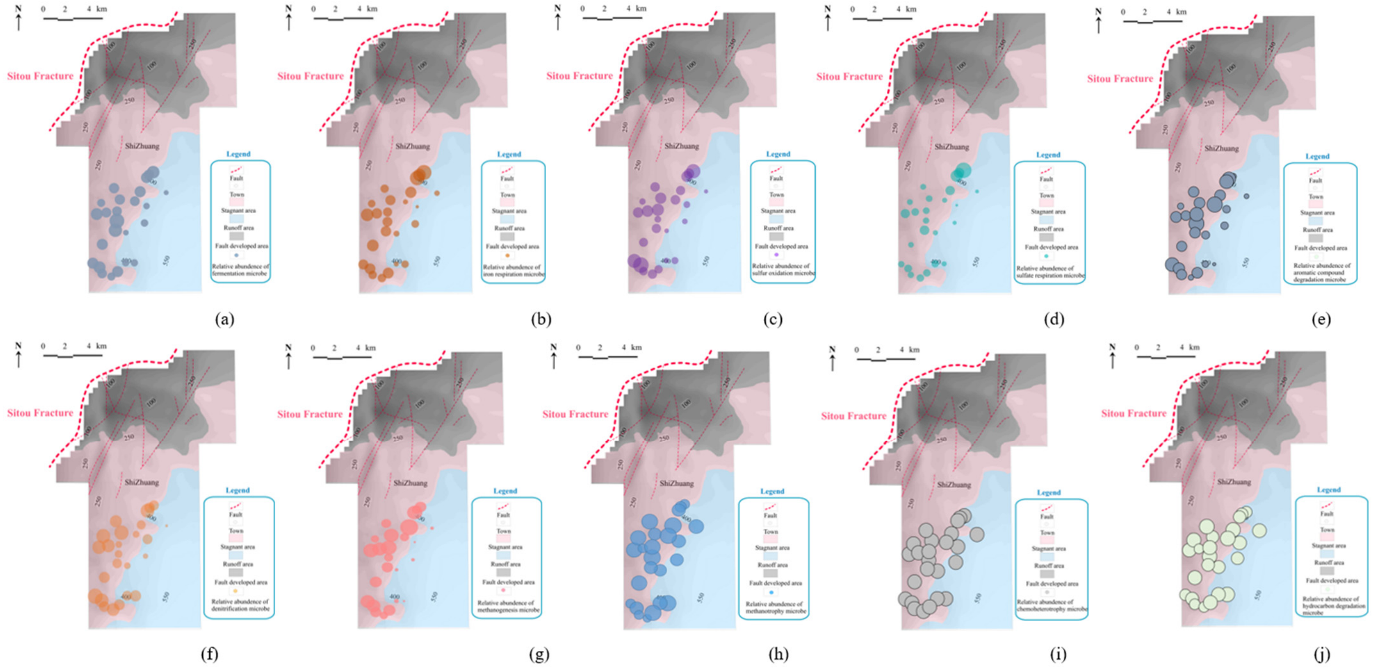

Figure S9. Bubble plot illustrating distribution characteristics of C-N-S microorganisms' abundance of water samples in the study area: (a) Fementation bacteria; (b) Iron respiration bacteria; (c) Sulfur oxidation bacteria; (d) Sulfate respiration bacteria; (e) Aromatic compound degradation bacteria; (f) Denitrification bacteria; (g) Methanogenesis archaea; (h) Methanotrophy; (i) Chemoheterotrophy bacteria; (j) Hydrocarbon degradation bacteria. The relative abundance of microorganisms was converted into a $\log_{10}$ (relative abundance*10^6) scale for better exhibition and comparison between runoff areas and stagnant areas. The area of the circle represents the abundance of different microorganisms. The base map divides the hydrological zones, different colors represent different hydrological zones, including runoff areas in the recharge zone, stagnant areas and deep coal areas.

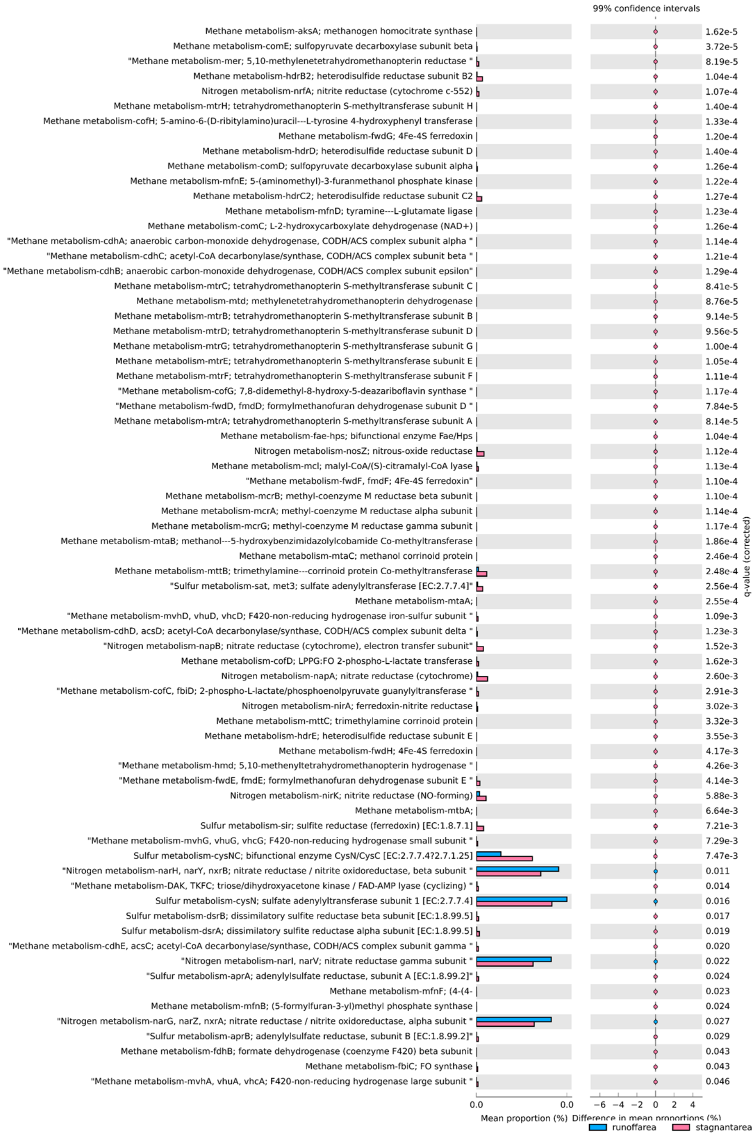
99% confidence intervals
Methane metabolism-aksA; methanogen homocitrate synthase 1.62e-5
Methane metabolism-comE; sulfopyruvate decarboxylase subunit beta 3.72e-5
"Methane metabolism-mer; 5,10-methylenetetrahydromethanopterin reductase " 8.19e-5
Methane metabolism-hdrB2; heterodisulfide reductase subunit B2 1.04e-4
Nitrogen metabolism-nrfA; nitrite reductase (cytochrome c-552) 1.07e-4
Methane metabolism-mtrH; tetrahydromethanopterin S-methyltransferase subunit H 1.40e-4
Methane metabolism-cofH; 5-amino-6-(D-ribitylamino)uracil---L-tyrosine 4-hydroxyphenyl transferase 1.33e-4
Methane metabolism-fwdG; 4Fe-4S ferredoxin 1.20e-4
Methane metabolism-hdrD; heterodisulfide reductase subunit D 1.40e-4
Methane metabolism-comD; sulfopyruvate decarboxylase subunit alpha 1.26e-4
Methane metabolism-mfnE; 5-(aminomethyl)-3-furanmethanol phosphate kinase 1.22e-4
Methane metabolism-hdrC2; heterodisulfide reductase subunit C2 1.27e-4
Methane metabolism-mfnD; tyramine---L-glutamate ligase 1.23e-4
Methane metabolism-comC; L-2-hydroxycarboxylate dehydrogenase (NAD+) 1.26e-4
"Methane metabolism-cdhA; anaerobic carbon-monoxide dehydrogenase, CODH/ACS complex subunit alpha " 1.14e-4
"Methane metabolism-cdhC; acetyl-CoA decarbonylase/synthase, CODH/ACS complex subunit beta " 1.21e-4
"Methane metabolism-cdhB; anaerobic carbon-monoxide dehydrogenase, CODH/ACS complex subunit epsilon" 1.29e-4
Methane metabolism-mtrC; tetrahydromethanopterin S-methyltransferase subunit C 8.41e-5
Methane metabolism-mtd; methylenetetrahydromethanopterin dehydrogenase 8.76e-5
Methane metabolism-mtrB; tetrahydromethanopterin S-methyltransferase subunit B 9.14e-5
Methane metabolism-mtrD; tetrahydromethanopterin S-methyltransferase subunit D 9.56e-5
Methane metabolism-mtrG; tetrahydromethanopterin S-methyltransferase subunit G 1.00e-4
Methane metabolism-mtrE; tetrahydromethanopterin S-methyltransferase subunit E 1.05e-4
Methane metabolism-mtrF; tetrahydromethanopterin S-methyltransferase subunit F 1.11e-4
"Methane metabolism-cofG; 7,8-didemethyl-8-hydroxy-5-deazariboflavin synthase " 1.17e-4
"Methane metabolism-fwdD, fmdD; formylmethanofuran dehydrogenase subunit D " 7.84e-5
Methane metabolism-mtrA; tetrahydromethanopterin S-methyltransferase subunit A 8.14e-5
Methane metabolism-fae-hps; bifunctional enzyme Fae/Hps 1.04e-4
Nitrogen metabolism-nosZ; nitrous-oxide reductase 1.12e-4
Methane metabolism-mcl; malyl-CoA/(S)-citramalyl-CoA lyase 1.13e-4
"Methane metabolism-fwdF, fmdF; 4Fe-4S ferredoxin" 1.10e-4
Methane metabolism-mcrB; methyl-coenzyme M reductase beta subunit 1.10e-4
Methane metabolism-mcrA; methyl-coenzyme M reductase alpha subunit 1.14e-4
Methane metabolism-mcrG; methyl-coenzyme M reductase gamma subunit 1.17e-4
Methane metabolism-mtaB; methanol---5-hydroxybenzimidazolylcobamide Co-methyltransferase 1.86e-4
Methane metabolism-mtaC; methanol corrinoid protein 2.46e-4
Methane metabolism-mttB; trimethylamine---corrinoid protein Co-methyltransferase 2.48e-4
"Sulfur metabolism-sat, met3; sulfate adenylyltransferase [EC:2.7.7.4]" 2.56e-4
Methane metabolism-mtaA; 2.55e-4
"Methane metabolism-mvhD, vhuD, vhcD; F420-non-reducing hydrogenase iron-sulfur subunit " 1.09e-3
"Methane metabolism-cdhD, acsD; acetyl-CoA decarbonylase/synthase, CODH/ACS complex subunit delta " 1.23e-3
"Nitrogen metabolism-napB; nitrate reductase (cytochrome), electron transfer subunit" 1.52e-3
Methane metabolism-cofD; LPPG:FO 2-phospho-L-lactate transferase 1.62e-3
Nitrogen metabolism-napA; nitrate reductase (cytochrome) 2.60e-3
"Methane metabolism-cofC, fbiD; 2-phospho-L-lactate/phosphoenolpyruvate guanylyltransferase " 2.91e-3
Nitrogen metabolism-nirA; ferredoxin-nitrite reductase 3.02e-3
Methane metabolism-mttC; trimethylamine corrinoid protein 3.32e-3
Methane metabolism-hdrE; heterodisulfide reductase subunit E 3.55e-3
Methane metabolism-fwdH; 4Fe-4S ferredoxin 4.17e-3
"Methane metabolism-hmd; 5,10-methenyltetrahydromethanopterin hydrogenase " 4.26e-3
"Methane metabolism-fwdE, fmdE; formylmethanofuran dehydrogenase subunit E " 4.14e-3
Nitrogen metabolism-nirK; nitrite reductase (NO-forming) 5.88e-3
Methane metabolism-mtbA; 6.64e-3
Sulfur metabolism-sir; sulfite reductase (ferredoxin) [EC:1.8.7.1] 7.21e-3
"Methane metabolism-mvhG, vhuG, vhcG; F420-non-reducing hydrogenase small subunit " 7.29e-3
Sulfur metabolism-cysNC; bifunctional enzyme CysN/CysC [EC:2.7.7.4 2.7.1.25] 7.47e-3
"Nitrogen metabolism-narH, narY, nxrB; nitrate reductase / nitrite oxidoreductase, beta subunit " 0.011
"Methane metabolism-DAK, TKFC; triose/dihydroxyacetone kinase / FAD-AMP lyase (cyclizing) " 0.014
Sulfur metabolism-cysN; sulfate adenylyltransferase subunit 1 [EC:2.7.7.4] 0.016
Sulfur metabolism-dsrB; dissimilatory sulfite reductase beta subunit [EC:1.8.99.5] 0.017
Sulfur metabolism-dsrA; dissimilatory sulfite reductase alpha subunit [EC:1.8.99.5] 0.019
"Methane metabolism-cdhE, acsC; acetyl-CoA decarbonylase/synthase, CODH/ACS complex subunit gamma " 0.020
"Nitrogen metabolism-narI, narV; nitrate reductase gamma subunit " 0.022
"Sulfur metabolism-aprA; adenylylsulfate reductase, subunit A [EC:1.8.99.2]" 0.024
Methane metabolism-mfnF; (4-(4- 0.023
Methane metabolism-mfnB; (5-formylfuran-3-yl)methyl phosphate synthase 0.024
"Nitrogen metabolism-narG, narZ, nxrA; nitrate reductase / nitrite oxidoreductase, alpha subunit " 0.027
"Sulfur metabolism-aprB; adenylylsulfate reductase, subunit B [EC:1.8.99.2]" 0.029
Methane metabolism-fdhB; formate dehydrogenase (coenzyme F420) beta subunit 0.043
Methane metabolism-fbiC; FO synthase 0.043
"Methane metabolism-mvhA, vhuA, vhcA; F420-non-reducing hydrogenase large subunit " 0.046
q-value (corrected)
0.0 0.0
−6 −4 −2 0 2 4
Mean proportion (%)
Difference in mean proportions (%)
runoffarea
stagnantarea

(a)

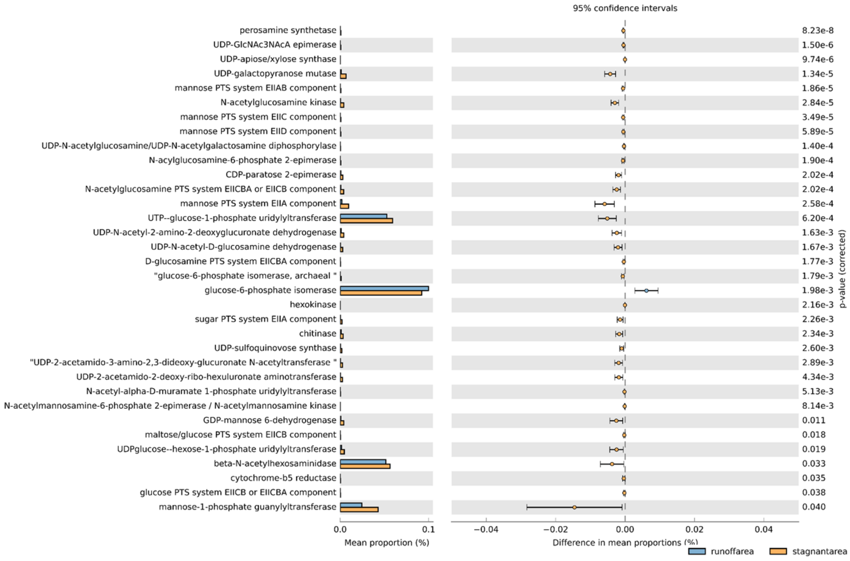
95% confidence intervals
perosamine synthetase 8.23e-8
UDP-GlcNAc3NAcA epimerase 1.50e-6
UDP-apiose/xylose synthase 9.74e-6
UDP-galactopyranose mutase 1.34e-5
mannose PTS system EIIAB component 1.86e-5
N-acetylglucosamine kinase 2.84e-5
mannose PTS system EIIC component 3.49e-5
mannose PTS system EIID component 5.89e-5
UDP-N-acetylglucosamine/UDP-N-acetylgalactosamine diphosphorylase 1.40e-4
N-acylglucosamine-6-phosphate 2-epimerase 1.90e-4
CDP-paratose 2-epimerase 2.02e-4
N-acetylglucosamine PTS system EIICBA or EIICB component 2.02e-4
mannose PTS system EIIA component 2.58e-4
UTP--glucose-1-phosphate uridylyltransferase 6.20e-4
UDP-N-acetyl-2-amino-2-deoxyglucuronate dehydrogenase 1.63e-3
UDP-N-acetyl-D-glucosamine dehydrogenase 1.67e-3
D-glucosamine PTS system EIICBA component 1.77e-3
"glucose-6-phosphate isomerase, archaeal " 1.79e-3
glucose-6-phosphate isomerase 1.98e-3
hexokinase 2.16e-3
sugar PTS system EIIA component 2.26e-3
chitinase 2.34e-3
UDP-sulfoquinovose synthase 2.60e-3
"UDP-2-acetamido-3-amino-2,3-dideoxy-glucuronate N-acetyltransferase " 2.89e-3
UDP-2-acetamido-2-deoxy-ribo-hexuluronate aminotransferase 4.34e-3
N-acetyl-alpha-D-muramate 1-phosphate uridylyltransferase 5.13e-3
N-acetylmannosamine-6-phosphate 2-epimerase / N-acetylmannosamine kinase 8.14e-3
GDP-mannose 6-dehydrogenase 0.011
maltose/glucose PTS system EIICB component 0.018
UDPglucose--hexose-1-phosphate uridylyltransferase 0.019
beta-N-acetylhexosaminidase 0.033
cytochrome-b5 reductase 0.035
glucose PTS system EIICB or EIICBA component 0.038
mannose-1-phosphate guanylyltransferase 0.040
p-value (corrected)
0.0 0.1
Mean proportion (%)
−0.04 −0.02 0.00 0.02 0.04
Difference in mean proportions (%)
runoffarea
stagnantarea

(b)

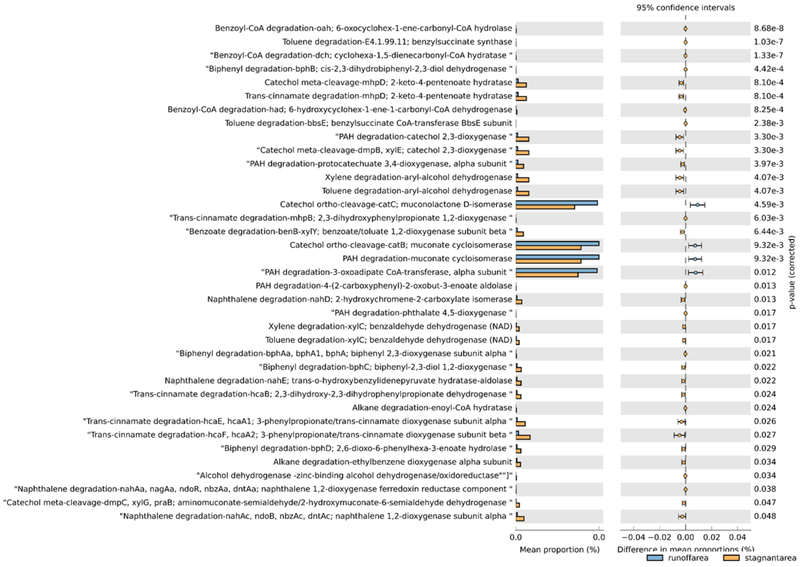

c)

Figure S10. Analysis of different relative abundances of functional genes between runoff and stagnant areas using Welch's *t*-test with FDR correction in STAMP (95 % confidence intervals, $p < 0.05$). (a) N-cycling genes, S-cycling genes and methane metabolism genes; (b) polysaccharide degradation gene; (c) Aromatic compounds degradation genes.

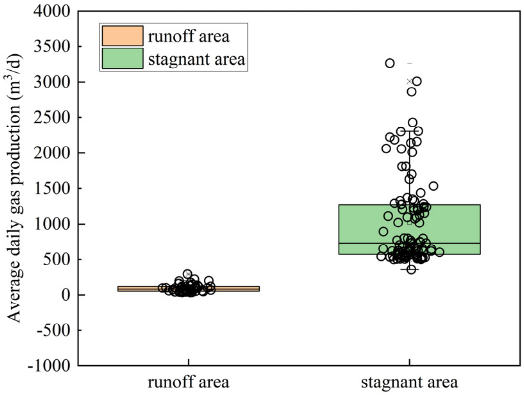

Figure S11. Comparison of average daily gas production from drainage wells in runoff and stagnant areas.

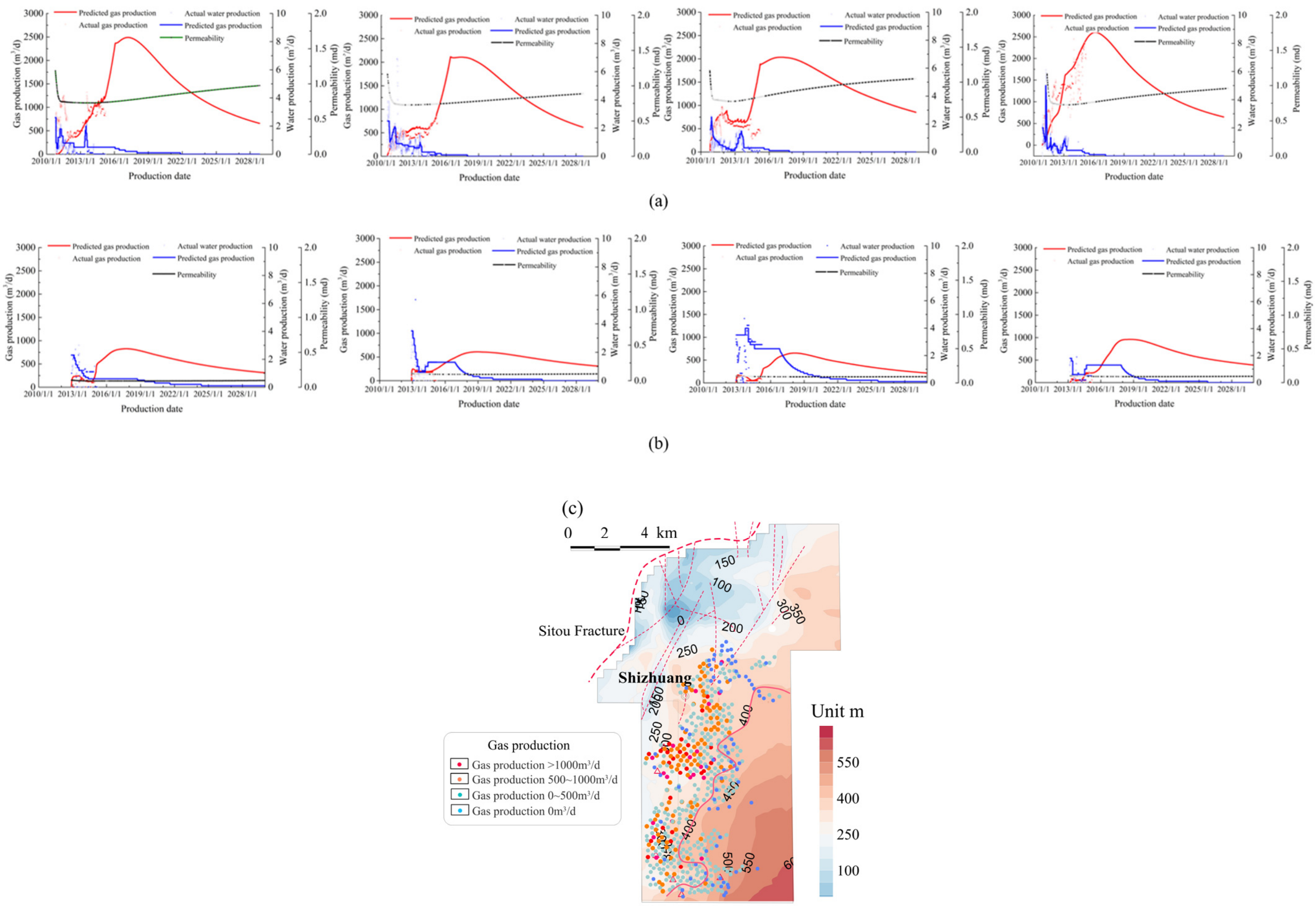

Figure S12. Numerical simulation of gas and water production in drainage wells in runoff area and stagnant area well group. (a) stagnant areas; (b) runoff areas; (c) Distribution of drainage wells in the Shizhuangnan block; the base map is the elevation contour of the #3 coal seam (m) and different drainage well colors represent the different daily gas productions, as shown in the legend.

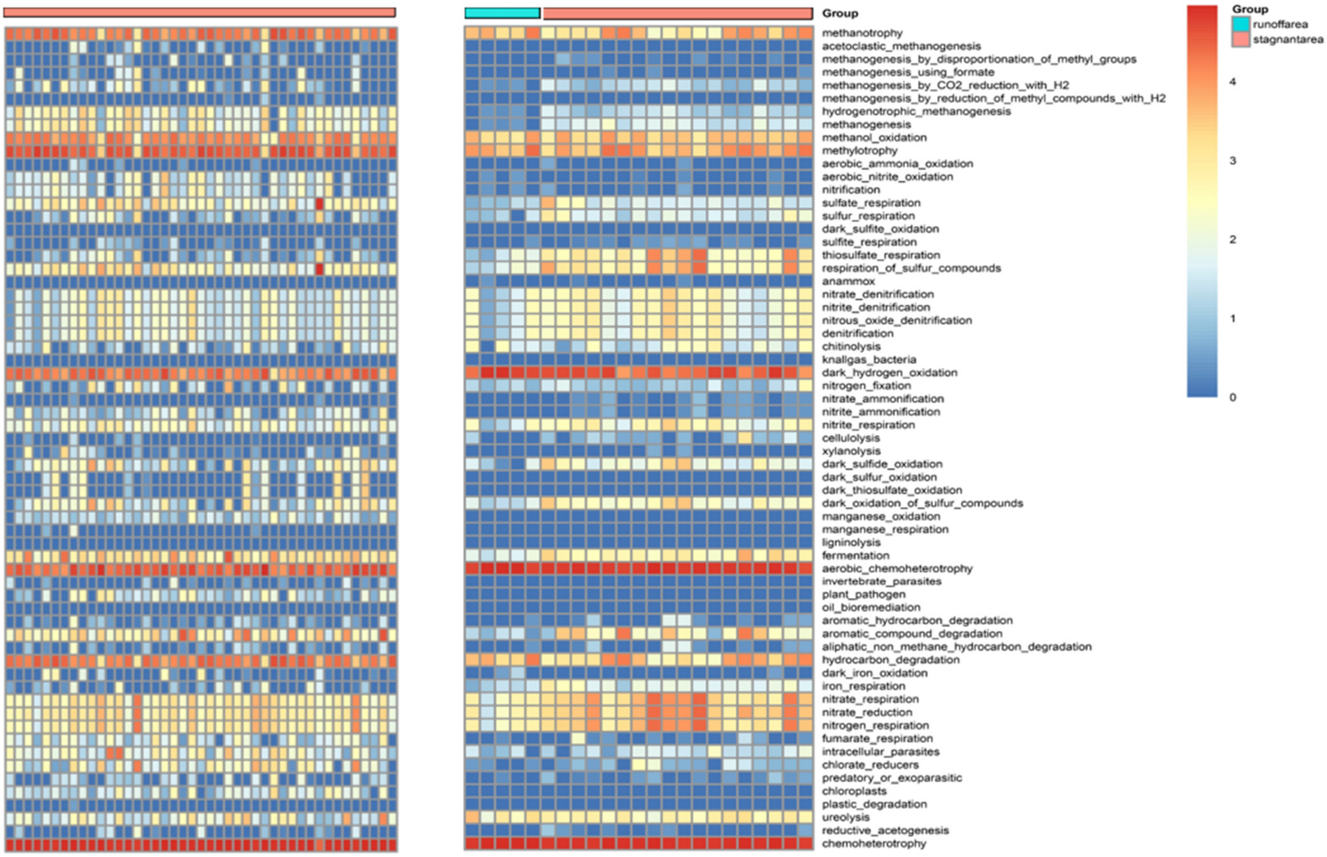

Figure S13. Heatmap showing relative abundance of C-N-S microorganisms of the 2016.12~ 2019.12 samples. The relative abundance of microorganisms was converted into a $\log_{10}$ (relative abundance*10^6) scale for better exhibition in the heatmap and the runoff and stagnant groups were distinguished in green and red, respectively.
